# Supplementary material for: Scalable analysis of multi-modal biomedical data
Source: Gigascience. 2021 Sep 11;10(9):giab058. doi: 10.1093/gigascience/giab058 (PMC8434767; doi:10.1093/gigascience/giab058)
Supplement: giab058_GIGA-D-20-00371_Revision_2 [file giab058_giga-d-20-00371_revision_2.pdf]

GigaScience

Scalable Analysis of Multi-Modal Biomedical Data

--Manuscript Draft--

**Manuscript Number:**	GIGA-D-20-00371R2
**Full Title:**	Scalable Analysis of Multi-Modal Biomedical Data
**Article Type:**	Technical Note
**Funding Information:**	Engineering and Physical Sciences Research Council (EP/M005852/1) — Dr. Michael Benedikt<br>Engineering and Physical Sciences Research Council (EP/R511742/1) — Dr. Michael Benedikt
**Abstract:**	Targeted diagnosis and treatment options are dependent on insights drawn from multi-modal analysis of large-scale biomedical datasets. Advances in genomics sequencing, image processing, and medical data management have supported data collection and management within medical institutions. These efforts have produced large-scale datasets and have enabled integrative analyses that provide a more thorough look of the impact of a disease on the underlying system. The integration of large-scale biomedical data commonly involves several complex data transformation steps, such as combining datasets to build feature vectors for learning analysis. Thus, scalable data integration solutions play a key role in the future of targeted medicine. Though large-scale data processing frameworks have shown promising performance for many domains, they fail to support scalable processing of complex datatypes. To address these issues and achieve scalable processing of multi-modal biomedical data, we present TraNCE, a framework that automates the difficulties of designing distributed analyses with complex biomedical data types. We outline research and clinical applications for the platform, including data integration support for building feature sets for classification. We show that the system is capable of outperforming the common alternative, based on "flattening" complex data structures, and runs efficiently when alternative approaches are unable to perform at all.
**Corresponding Author:**	Jaclyn Smith<br>University of Oxford<br>Oxford, Oxfordshire UNITED KINGDOM
**Corresponding Author Secondary Information:**	
**Corresponding Author's Institution:**	University of Oxford
**Corresponding Author's Secondary Institution:**	
**First Author:**	Jaclyn Smith
**First Author Secondary Information:**	
**Order of Authors:**	Jaclyn Smith<br>Yao Shi<br>Michael Benedikt<br>Milos Nikolic
**Order of Authors Secondary Information:**	
**Response to Reviewers:**	All minor revisions have been made to the pdf manuscript.
**Additional Information:**	
**Question**	**Response**
Are you submitting this manuscript to a special series or article collection?	No

Powered by Editorial Manager® and ProduXion Manager® from Aries Systems Corporation

**Experimental design and statistics**<br><br>Full details of the experimental design and statistical methods used should be given in the Methods section, as detailed in our Minimum Standards Reporting Checklist. Information essential to interpreting the data presented should be made available in the figure legends.<br><br>Have you included all the information requested in your manuscript?	Yes
**Resources**<br><br>A description of all resources used, including antibodies, cell lines, animals and software tools, with enough information to allow them to be uniquely identified, should be included in the Methods section. Authors are strongly encouraged to cite Research Resource Identifiers (RRIDs) for antibodies, model organisms and tools, where possible.<br><br>Have you included the information requested as detailed in our Minimum Standards Reporting Checklist?	Yes
**Availability of data and materials**<br><br>All datasets and code on which the conclusions of the paper rely must be either included in your submission or deposited in publicly available repositories (where available and ethically appropriate), referencing such data using a unique identifier in the references and in the "Availability of Data and Materials" section of your manuscript.<br><br>Have you have met the above requirement as detailed in our Minimum Standards Reporting Checklist?	Yes

Manuscript
Click here to access/download;Manuscript;manuscript-R1-edits.pdf

Placeholder for OUP logo oup.pdf

Placeholder for journal logo gigascience-logo.pdf

GigaScience, 2017, 1–17

doi: xx.xxxx/xxxx
Manuscript in Preparation
Paper

PAPER

Scalable Analysis of Multi-Modal Biomedical Data

Jaclyn Smith[1]*, Yao Shi[1], Michael Benedikt[1] and Milos Nikolic[2]

[1]University of Oxford and [2]University of Edinburgh

jaclyn.smith@cs.ox.ac.uk, yao.shi@kellogg.ox.ac.uk, michael.benedikt@cs.ox.ac.uk, milos.nikolic@ed.ac.uk

Targeted diagnosis and treatment options are dependent on insights drawn from multi-modal analysis of large-scale biomedical datasets. Advances in genomics sequencing, image processing, and medical data management have supported data collection and management within medical institutions. These efforts have produced large-scale datasets and have enabled integrative analyses that provide a more thorough look of the impact of a disease on the underlying system. The integration of large-scale biomedical data commonly involves several complex data transformation steps, such as combining datasets to build feature vectors for learning analysis. Thus, scalable data integration solutions play a key role in the future of targeted medicine. Though large-scale data processing frameworks have shown promising performance for many domains, they fail to support scalable processing of complex datatypes. To address these issues and achieve scalable processing of multi-modal biomedical data, we present *TraNCE*, a framework that automates the difficulties of designing distributed analyses with complex biomedical data types. We outline research and clinical applications for the platform, including data integration support for building feature sets for classification. We show that the system is capable of outperforming the common alternative, based on "flattening" complex data structures, and runs efficiently when alternative approaches are unable to perform at all.

Key words: Nested data; Distributed processing; Spark; Query compilation; Multi-omics analysis; Multi-modal data integration;

Background

The affordability of genomic sequencing, the advancement of image processing, and the improvement of medical data management have made the biomedical field an interesting application domain for integrative analyses of complex datasets. Targeted medicine is a response to these advances, aiming to tailor a medical treatment to an individual based on their genetic, lifestyle, and environmental risk factors [20]. Analyses that combine molecular measurements from multi-omics data provide a more thorough look at the disease at hand, and the relative impacts on the underlying system; thus, the reliability of such targeted treatments is dependent on multi-modal, cohort-based analyses.

Targeted medicine has improved data management and data collection within medical institutions, which are now capable of producing biomedical datasets at outstanding rates. For example, the sequence archive from the NIH has exhibited exponential growth in less than a decade [19]. In addition, these efforts have also spurred consortium dataset collection and biobanking efforts [9]. These are consolidated data sources from hundreds-of-thousands of patients and counting, such as 1000 Genomes [4], International Cancer Genome Consortium (ICGC) [23], The Cancer Genome Atlas (TCGA) [55], and UK BioBank [48]. This scenario has introduced a demand for data processing solutions that can handle such large-scale datasets; thus, scalable data integration and aggregation solutions capable of supporting joint inference play a key role in advancing biomedical analysis.

Modern biomedical analyses are pipelines of data access mechanisms and analytical components that operate on and produce datasets in a variety of complex, domain-specific formats. Integrative analyses of complex datasets can bring many challenges, which are compounded with large-scale data. These challenges can be related to performance or programming issues. Performance issues arise because distribution strategies are not favorable for nested datasets, often hindering parallel execution and exhibiting poor resource utilization. Programming issues arise when associations must be made on nested attributes, making implementation not straightforward. To understand these challenges, we will now introduce a running example and use that example to overview multi-omics analysis, distributed processing systems, and the challenges that arise when these two worlds collide.

Running example. Cancer progression can be determined by the accumulation of mutations and other genomic aberrations within a sample [8]. Consider an integrative, multi-omics analysis that aims to identify driver genes in cancer based on mutational effects and the abundance of gene copies in a sample [60]. This analysis combines single-point, somatic mutations and gene-level copy number information to calculate a likelihood score that a candidate gene is a driver within each sample, known as a hybrid score. *Candidate genes* are assigned to mutations based on the proximity of a mutation to a gene. In a naive assignment, candidacy is established if the mutation lies directly on a gene; however, mutations have been shown to form long-range functional connections with genes [40], so candidacy can best be assigned based on a larger flanking region of the genome.

Multi-omics datasets. To understand the complexities of such an integrative analysis, first consider the data sources involved. The Genomic Data Commons (GDC) [15] provides public access to clinical information (`Samples`), somatic mutation occurrences (`Occurrences`), and copy number variation (`CopyNumber`). Assume access to each of these data sources returns a collection of objects in JSON (JavaScript Object Notation), a popular format for nested data, where [ ] denotes a collection type and { } denotes an object type [37].

The `Samples` data source returns metadata associated with cancer samples. A simplified version of the schema contains a sample identifier (`sid`) and a single attribute `tumorsite` that specifies the site of tumor origin; the type of `Samples` is

$$[\,\{\, \texttt{sid} : \textit{string}, \texttt{tumorsite} : \textit{string} \,\}\,]. \qquad (1)$$

The copy number variation (CNV) data source returns by-gene copy number information for each `sid`; this is the number of copies of a particular gene

Key Points

- Modern biomedical analyses are integrated pipelines of data access mechanisms and analysis components that operate on and produce datasets in a variety of complex, domain specific formats.
- Scalable data integration and aggregation solutions that support joint inference on such large-scale datasets play a key role advancing biomedical analysis.
- Query compilation techniques that optimize nested data processing are essential for scaling multi-modal, biomedical analysis.

measured in a sample. The type of copy number information is:

$$[\,\{\,\texttt{sid} : \textit{string}, \texttt{gene} : \textit{string}, \texttt{cnum} : \textit{int}\,\}\,]. \tag{2}$$

The `Occurrences` data source contains somatic mutations and associated annotation information for each sample. An occurrence is a single, annotated mutation belonging to a single sample. The type of `Occurrences` is:

$$\begin{aligned}&[\,\{\,\texttt{sid} : \textit{string}, \texttt{contig} : \textit{string}, \texttt{start} : \textit{int}, \texttt{end} : \textit{int},\\ &\quad \texttt{reference} : \textit{string}, \texttt{alternate} : \textit{string}, \texttt{mutationId} : \textit{string},\\ &\qquad \texttt{candidates} : [\,\{\,\texttt{gene} : \textit{string}, \texttt{impact} : \textit{string},\\ &\qquad\quad \texttt{sift} : \textit{real}, \texttt{poly} : \textit{real},\\ &\qquad\quad \texttt{consequences} : [\,\{\,\texttt{conseq} : \textit{string}\,\}\,]\,\}\,]\,\}\,].\end{aligned} \tag{3}$$

The attribute `candidates` identifies a collection of objects that contain attributes corresponding to the predicted effects a mutation has on a gene; i.e. *variant annotations* sourced from the Variant Effect Predictor (VEP) [32]. The `impact`, `sift`, `poly`, and `conseq` attributes provide impact scores denoting estimated consequence a mutation has to a gene based on sequence conservation, predicted functional changes [1, 52], and sequence ontology (SO) terms [11].

The `Samples` and `CopyNumber` data source types map perfectly into a relational scenario, such as a table in SQL (Structured Query Language) or a DataFrame in Pandas [51]. With all attributes of scalar type (integer, string, etc.), these data sources are considered *flat*. The `Occurrences` data source has a nested collection `candidates` on the first level and another nested collection `consequences` on the second level. When a collection has attributes of collection type, it is referred to as a *complex value* or a *nested collection*.

In the running example, the gene-based copy number values need to be combined with the mutational impact values. This requires associating the flat `CopyNumber` dataset with the `Occurrences` dataset based on `sid` and `gene`. The impact measurements and copy number values are then combined and a collection of candidate genes with corresponding hybrid scores are returned after summing across the unique genes for each sample. The output type of the running example is thus:

$$[\,\{\,\texttt{sid} : \textit{string}, \texttt{scores} : [\,\{\,\texttt{gene} : \textit{string}, \texttt{score} : \textit{real}\,\}\,]\,\}\,]. \tag{4}$$

The nested nature of this analysis is further complicated when the data is large and distributed computing is needed.

Distributed processing frameworks. Large-scale, distributed data processing platforms, such as Apache Spark [59], have become indispensable tools for modern data analysis. The wide adoption of these platforms stems from powerful functional-style APIs (Application Programming Interface) that allow programmers to express complex analytical tasks while abstracting distributed resources and data parallelism. Despite natively supporting nested data, distribution strategies often fail to process nested collections at scale, especially for a small number of top-level tuples or large inner collections. Further, data scientists who work on local analysis pipelines often have difficulties translating analyses into distributed settings.

Distributed processing frameworks work on top of a cluster of machines where one is designated as the central, or *coordinator* node, and the other nodes are *workers*. Figure 1 shows the setup of a Spark cluster; an application is submitted to the coordinator node, which then delegates tasks to worker nodes in a highly distributed, parallel fashion. A user never communicates with a worker node directly. A distributed processing API communicates high-level analytical tasks

Compiled on: Friday 30th July, 2021.
Draft manuscript prepared by the author.

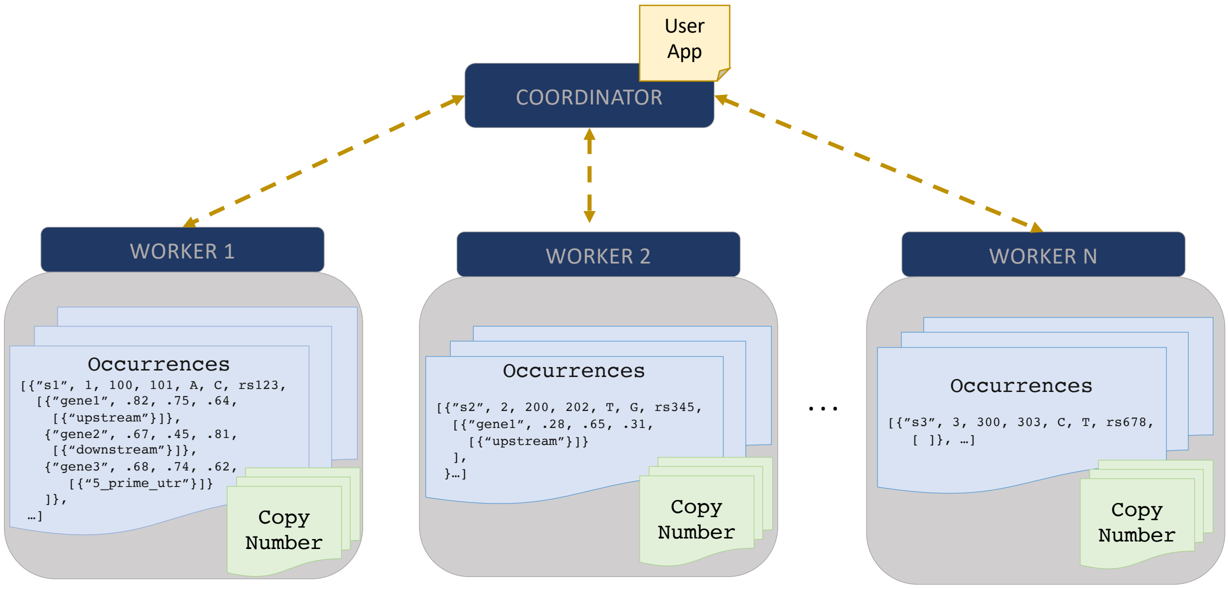

Figure 1. Set up of a Spark cluster with distributed representation of `Occurrences` and `CopyNumber` cached in memory across N worker nodes. User applications are submitted to the coordinator, which delegates tasks to the worker nodes to support distributed execution. Figure 2 is an example of a user application.

```
1 case class FlatOccurrence(sid: String, gene: String,
      impact: Double)
2
3 Occurrences.flatMap(o =>
4   o.candidates.map(t =>
5     FlatOccurrence(o.sid, t.gene, t.impact))
6 ).join(CopyNumber, Seq("sid", "gene"))
7  .groupByKey(s => s.sid)
```

Figure 2. Example Spark application that groups somatic mutations and copy number information by sample.

to the coordinator while abstracting data distribution and task delegation from the user.

Spark uses a specialized data structure for representing distributed data, where a *partition* is the smallest unit of distribution. When a flat data source, such as `Samples` and `CopyNumber`, is imported into Spark each item of the collection is allocated in round-robin fashion to each partition. The same import strategy is applied to nested dataset, with the nested attributes persisting in the same partition as their parent; we refer to this as a *top-level distribution strategy*. Figure 1 displays how `Occurrences` would be stored in memory across worker nodes, distributing top-level objects with `candidates` and `consequences` nested within the same location.

Spark provides an API for performing batch operations over distributed collections. Figure 2 presents a Scala program that uses the Spark API to associate `CopyNumber` to the relevant gene and samples in `Occurrences`; this is the association required for the running example.

The program starts by defining a case class, named `FlatOccurrence`, which encapsulates objects of type [{ `sid` : *string*, `gene` : *string*, `impact` : *real* }]. The `flatMap` operation (lines 3-5) works locally at each partition, iterating over top-level objects in `Occurrences` and navigating into `candidates` to create instances of `FlatOccurrence` objects. The `join` operator (line 6) merges tuples from the result of `flatMap` and `CopyNumber` based on the equality of (`sid`, `gene`) values; these are the keys of a *key-based partitioning guarantee* that sends all matching values to the same partition. The process of moving data to preserve a partitioning guarantee is known as *shuffling*. The final `groupByKey` operation (line 7) groups the joined result based on unique `sid` values; this is a key-based operation that sends all tuples with matching `sid` values to the same partition, producing a final output type of: [{ `sid` : *string*, [{ `gene` : *string*, `impact` : *real*, `cnum` : *int* }] }].

Given the complexities of biomedical analyses and the related aspects of distributed computing frameworks described above, we now detail the performance and programming challenges that arise when implementing analyses over distributed, nested collections.

Challenges of distributed, multi-omics analyses. *Performance issues* are rooted in the top-level distribution and key-based partitioning strategies of distributed processing systems. First, few top-level values can hinder distribution strategies for nested data. For example, the `groupByKey` operation in Figure 2 for a dataset with a small number of samples, such as the 51 samples of the TCGA lymphoma dataset, will distribute objects across no more than 51 partitions. This is poor resource utilization for a cluster that supports more partitions. Second, large inner collections, such as the collective copy number information for every gene, can overwhelm the physical storage of a partition. This leads to time-consuming processes of moving values in and out of memory. Both of these performance issues can lead to *skew* related bottlenecks that make certain tasks run considerably longer than others.

Programming issues arise when joining on a nested attribute, such as the join between `Occurrences` and `CopyNumber` in the running example. After preprocessing, the association on the nested attributes of `Occurrences` can be performed with a join in SQL, Pandas, or Spark as we have seen in Figure 2; however, the preprocessing required is not straightforward for complex datasets.

Since `Occurrences` is distributed, the `gene` join keys are nested within each partition, and are not directly accessible without iterating inside the nested collections. Even an iteration inside `candidates` cannot directly perform the conditional join filter on `CopyNumber` because it is itself distributed. An attempt to reference a distributed resource within a transformation of another distributed resource will result in error because a single partition is not aware of the other distributed resources and has no power to delegate tasks to workers. The solution is to replicate `CopyNumber` to each worker node, which can be too expensive, or rewrite to flatten `Occurrences` and bring `gene` attributes to the top-level, which can lead to exponential blowups. Flattening can also yield incorrect results, due to empty nested collections and the loss of relationship information between nested child objects and their parent attributes. For example, simply applying the `flatMap` in Figure 2 does produce a flat bag from a nested one. But it will lose all occurrences that have empty `candidates` collections, which may make certain operations on the nested object impossible to retrieve from the output. The `flatMap` example from the figure also illustrates the performance issues associated with flattening. Even though it loses some information, flattening the 8000 top-level records of the TCGA lymphoma `Occurrences` dataset produces 250000 records, which is a 31x increase. Further, regrouping this information based on the unique `oid` identifier returns only 1700 top-level tuples since we have lost occurrences with empty collections. In general, manual implementations of flattening procedures that perform adequately and ensure correctness are non-trivial [13].

Related work. A wide range of tools are available to assist biological analyses. Workflow engines ease the process of connecting many external software systems while producing repeatable analyses; examples include Galaxy [2], Cromwell [53], Arvados [10], and Taverna [36]. Corresponding workflow languages describe imperative pipelines requiring manual optimizations to each individual pipeline component. In contrast, high-level, declarative languages better insulate pipeline writers from platform details, while also providing the ability to leverage database-style query compilation and query optimization techniques. Many genomic-specific languages have been developed that target distributed processing platforms, such as GenoMetric [30], Hail [18], Adam [31, 35], and Glow [16]. These provide advantages for a particular class of transformations, but would not suffice for pipelines that integrate a variety of relational and nested data types.

TraNCE is introduced in [43]. That paper presents the core shredding and skew-handling techniques, and provides micro-benchmarks that show the impact of their performance relative to the baseline, standard pipeline and to external competitors. The arxiv paper [41] and github repository [42] associated with [43] provide additional comparisons. The focus of this work is the use of the tool in the context of biology and the remaining architectural pieces that enable that use, including the interaction of the language with statistical libraries, notebook environments, and extended skew-handling techniques.

Proposed solution. To address these issues and achieve scalable, distributed processing of multi-modal biomedical data, we propose *TraNCE* (Transforming Nested Collections Efficiently). *TraNCE* is a compilation framework that automates the difficulties of designing distributed analyses with complex, biomedical data types. The framework provides a high-level language suitable for users of varying levels of data science expertise, providing an abstraction to the difficulties of integrating complex datasets and programming with a distributed collection API. The system uses query compilation and optimization techniques to ease the difficulty of handling nested collections, and is designed for arbitrary, multi-modal analyses of complex data types.

The paper proceeds as follows. The Methods section outlines the components of the *TraNCE* platform, describing the major components by means of example. We overview several omics-based use cases that have been trialed with our framework, including performance metrics in the Results section. Finally, we conclude with a summary of contributions and future work.

Methods

TraNCE platform

TraNCE [RRID:SCR_021252] is a compilation framework that transforms declarative programs over nested collections into distributed execution plans. This section discusses several key aspects of the platform, including program compilation, program and data shredding, and skew-resilience. *Program compi-*

lation leverages a high-level, declarative source language that allows users to describe programs over nested collections. The framework insulates the user from the difficulties of handling nested collections in distributed environments.

Two compilation routes are provided, standard and shredded, that apply optimizations while transforming input programs into executable code. *Standard compilation* uses unnesting [13] techniques to apply optimal flattening methods in order to compute on nested values. This compilation route automatically handles introducing NULL values and unique identifiers that preserve correctness. The standard compilation acts as a baseline for the *shredded compilation*; this compilation route is reflective of current procedures to handle nested data, such as what is provided in Spark-SQL [3] [RRID:SCR_016557]. The shredded route optimizes the standard route with *shredding* techniques that transform a program operating on nested collections into a collection of programs that operate over flat collections [6, 56], thus enabling parallelism beyond top-level records.

The result of each compilation route is an Apache Spark program that is suited for distributed execution. We apply dynamic optimizations at runtime that overcome skew-related bottlenecks. *Skew-resilience* prevents the overloading of a partition at anytime during the analysis to avoid such bottlenecks in execution, and maintain better overall distribution of the data.

Figure 3 provides a schematic of the framework, including interaction with a Spark cluster for the shredded compilation method. The Spark cluster setup for the standard compilation method is the classic setup depicted in Figure 1.

The next sections overview each of the framework components, drawing specific attention to multi-omics analysis. We begin with an introduction to the *TraNCE* language, and then describe the standard compilation and shredded compilation routes. Finally, we detail the skew-resilient processing optimization and the code generation process.

High-level Language

TraNCE provides a language for describing biomedical analyses as high-level collection programs; this language is a variant of nested relational calculus (NRC) [5, 56]. Here we provide a walk-through of the language using several example programs over the `Occurrences`, `CopyNumber`, and `Samples` data sources. We will introduce some basic aspects of the language, eventually leading up to the program associated with the running example. The full syntax of the TraNCE language is provided in [43].

TraNCE programs operate on collections of objects. Objects are tuples of values for a fixed set of attributes, with all objects of one collection having the same type. Attributes can be of basic scalar type (integer, string, etc.) or of collection type, thus providing support for nested data. We denote collection types with [ ] and object types with { } to follow JSON syntax. For example, the type of `Occurrences` at (3) is a collection that itself contains collections, with `candidates` and `consequences` corresponding to collection types and all other attributes as scalars.

The main advantage of the *TraNCE* language is the ability to manipulate nested collections and return results with nested output type, while abstracting out the complications of nested data distribution from the user. Consider the following program, assigned to `OccurrProj` via the ⇐ operator, that requests only specific attributes from the `Occurrences` data source:

```
OccurrProj ⇐
for o in Occurrences union
 [{sid := o.sid, candidates :=
  for t in o.candidates union
   [{gene := t.gene, impact := t.impact}]}]
```

The `OccurrProj` program iterates over `Occurrences` persisting only the attributes required for the running example. The program first iterates over the top-level of `Occurrences`, preserving only the `sid` attribute, and creates a nested `candidates` collection by iterating over the `candidates` collection and preserving the `gene` and `impact` attributes. Note that this is similar to lines 3-5 from Figure 2; however, this program follows the nested structure of `Occurrences` and does no flattening.

The next step of the running example is to define the association between `CopyNumber` and `Occurrences`. The language allows one to specify such associations between data sources and on nested attributes without explicitly defining a flattening operation. For example, the `OccurCNV` program associates copy number information based on both the top-level `sid` attribute and the nested `gene` attribute of `Occurrences`.

```
OccurCNV ⇐
for o in Occurrences union
 [{sid := o.sid, candidates :=
  for t in o.candidates union
   for c in CopyNumber union
    if o.sid == c.sid && t.gene == c.gene then
     [{gene := t.gene, impact := t.impact, cnum := c.cnum}]}]
```

The `OccurCNV` program iterates over `Occurrences` following its original structure. The iteration over `CopyNumber` is specified in the second level, providing immediate access to the nested `gene` attribute and allowing the user to define an association between `sid` and `gene`. The result returns the original structure of the first two levels of `Occurrences`, annotating each of the candidate genes for every mutation with the relevant copy number information.

Standard arithmetic operations and built-in support for aggregation are provided in the language. The $\texttt{sumBy}_{key}^{value}(e)$ function can be used for counting and summing based on a unique key. The *key* and *value* parameters can reference any number of attributes from the input expression *e*. `sumBy` can be applied at a specific level as long as the input *e* has no nesting. For example, the `OccurAgg` program performs the association from the running example, while also summing over the product of the respective copy number and mutational impact values for every sample in occurrences.

```
OccurAgg ⇐
for o in Occurrences union
 [{sid := o.sid, candidates :=
  sumBy^score_gene(
  for t in o.candidates union
   for c in CopyNumber union
    if o.sid == c.sid && t.gene == c.gene then
     [{gene := t.gene, score := t.impact * c.cnum}])}]
```

The `OccurAgg` program extends the previous programs, returning the sum of the product of copy number and variant information based on the unique genes in `candidates`. The `sumBy` is applied to the first level of `Occurrences` with `gene` as *key* and `score` as *value*. With all attributes of scalar type, the input corresponding to *e* has a flat type [{ `gene` : *string*, `score` : *real* }].

All programs so far have followed the structure of the `Occurrences` data source, grouping the genes associated to each mutation and each sample. If the goal is to create candidate gene collections per sample, then we can additionally group by sample using the $\texttt{groupBy}_{key}(e)$ function. The following is the full program that represents the running example, which we denote `SGHybridScores`; this program creates hybrid-scores for each sample, summing the combination of annotation information and copy number information across all candidate genes for all mutations associated to the top-level sample.

```
SGHybridScores ⇐
groupBy^gene,score_sid(
 sumBy^score_sid,gene(
  for o in Occurrences union
   for t in o.candidates union
    for c in CopyNumber union
     if o.sid == c.sid && t.gene == c.gene then
      [{sid := o.sid, gene := t.gene,
       score := t.impact * (c.cnum + 0.01) * t.sift * t.poly}]))
```

The expression inside of `sumBy` captures the navigation into `candidates`, associating each candidate gene at this level with `CopyNumber` on the `gene` and `sid` attribute. The product of all these measurements produces the intermediate score for each of the candidate genes within the `candidates` collection for each mutation. The final hybrid-scores are calculated by aggregating all intermediate scores across all mutations within a sample, returning a hybrid-score associated to each unique gene for each sample using `sumBy`. The result of

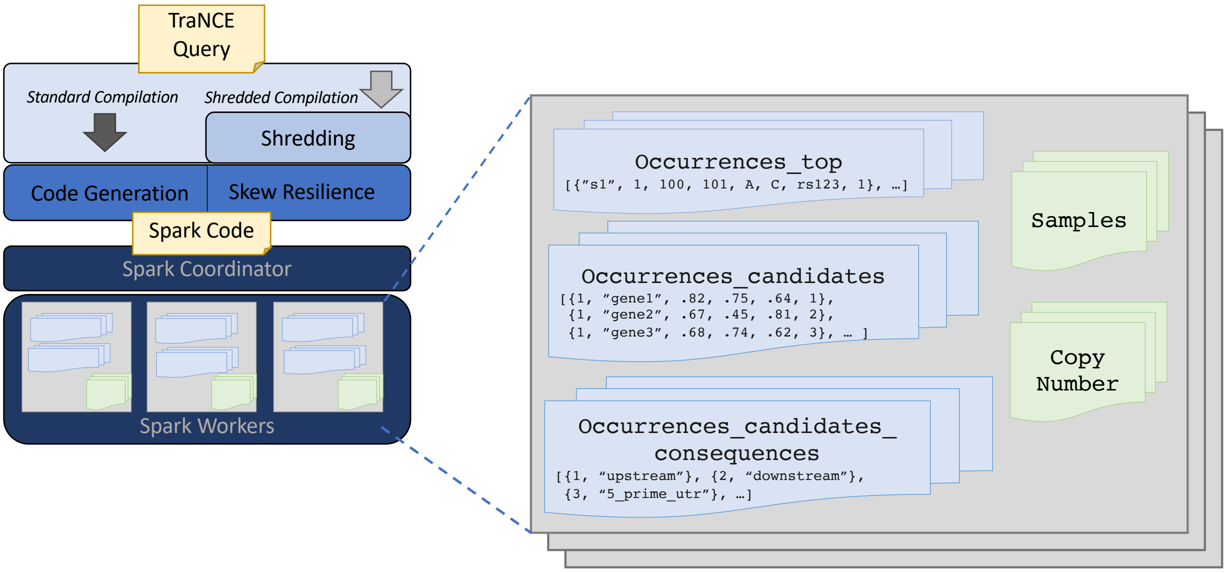

Figure 3. System architecture of *TraNCE*, presenting two compilation routes that result in executable code. The Spark cluster provides a schematic representation of the shredded compilation route, where the shredded inputs of `Occurrences` are cached in memory across worker nodes.

this aggregation is further grouped with `groupBy` to return the hybrid scores associated to every sample, producing output type:

$$[\,\{\,\texttt{sid} : \textit{string}, \texttt{scores} : [\,\{\,\texttt{gene} : \textit{string}, \texttt{score} : \textit{real}\,\}\,]\,\}\,].$$

The next program extends upon the running example `SGHybridScores` program, grouping the sample-grouped hybrid scores by tumor site using the `Samples` table. We denote this program as `TGHybridScores`:

```
TumorSites ⇐
 dedup(for s in Samples union
  [{tumorsite := s.tumorsite}])

TGHybridScores ⇐
for t in TumorSites union
 [{tumorsite := t.tumorsite, samples :=
  for s in Samples union
   if t.tumorsite == s.tumorsite then
    [{sid := s.sid, scores := sumBy^score_gene(
     for o in Occurrences union
      if s.sid == o.sid then
       for a in o.candidates union
        for c in CopyNumber union
         if o.sid == c.sid && a.gene == c.gene then
          [{gene := t.gene, score :=
            t.impact * (c.cnum + 0.01) * t.sift * t.poly}])}]}]
```

The tumor-grouped hybrid score program first iterates over the `Samples` data source to create a unique set of tumor sites with `dedup`. `dedup` is a function that returns a collection with all duplicates removed. The second part of the program `TGHybridScores` iterates over these unique groups to create top-level groupings based on `tumorsite`. The program then enters the first level at `samples` where it proceeds to iterate over `Samples` creating first-level groupings based on `sid`. The second level begins at `scores` which performs the `sumBy` aggregation described in the previous program. The result of the `TGHybridScores` program is every sample-based hybrid score further grouped by tumor site with the output type:

$$[\,\{\,\texttt{tumorsite} : \textit{string}, \texttt{samples} : [\,\{\,\texttt{sid} : \textit{string}, \texttt{scores} : [\,\{\,\texttt{gene} : \textit{string}, \texttt{score} : \textit{real}\,\}\,]\,\}\,]\,\}\,].$$

Note that the first portion of `TGHybridScores` that creates a unique set of tumor sites, and then iterates over then to create distinct top-level keys with nested groups is the exact operation as *groupBy*; thus the *groupBy* operation is a function that enables users to describe such key-based grouping operations in a more concise way. The rest of the examples in this section will refer to the extended running example `TGHybridScores` in order to describe the compilation routes with the base syntax of the language.

The *TraNCE* programs described in this section highlight the advantages of using a variation of NRC to describe analyses over nested collections. Each analysis is similar to pseudo-code where the user describes actions on nested collections, without considering implementation details that are specific to a distributed environment.

Standard compilation

The standard compilation route translates *TraNCE* programs into executable Spark applications, while handling the difficulties of flattening procedures. Figure 3 provides a high-level schematic of the standard pipeline. This compilation is based on unnesting techniques that automate the flattening process by automatically inserting NULL and unique identifiers (ID) to preserve correctness [13]. The unnesting process starts from the outermost level of a program, recursively defining a Spark execution strategy. A new nesting level is entered when an object contains an expression of collection type. Before entering the new nesting level, a unique ID is assigned to each object at that level. At each level, the process maintains a set of attributes, including the unique IDs, to use as the prefix for the key in grouping and `sumBy` operations.

Consider running the standard compilation for the `TGHybridScores` analysis. The Spark application generated for this program starts by iterating over `Occurrences` values, flattening each of the nested items inside `candidates` with `flatMap`. Prior to this, `Occurrences` is indexed to ensure tracking of top-level objects. If `candidates` is an empty collection, lower level attributes exist as null values. The result of flattening has `gene` attributes that are accessible at top level. The flattened result is joined with `CopyNumber` based on `sid` and `gene` attributes, and the product associated to `score` is calculated. This result is further joined with `Samples` and grouped by sample using the `groupByKey` operation. A final call to `groupByKey` groups again by `tumorsite` to produce the final result.

Projections are pushed throughout the execution strategy, ensuring that only used fields are persisted. The framework can also introduce intermediate aggregations, such as combining `impact`, `sift`, and `poly` in `Occurrences` prior to joining with `CopyNumber`.

The standard compilation route is the baseline for processing nested queries, generating execution strategies that are reflective of the state-of-the-art flatten-

ing procedures in current systems capable of processing nested queries. We have previously shown that due to additional optimizations we apply, such as intermediate aggregations, the standard route has better performance in relation to classical flattening methods [43]. Nonetheless, flattening methods do not scale, so the standard compilation route is provided as the basis for the scalable shredded compilation route.

Shredded compilation

The shredded compilation route takes the same high-level *TraNCE* program as in the standard route, extending compilation to support a more succinct data representation. Analytics pipelines, regardless of final output type, produce intermediate nested collections that can be important in themselves: either for use in multiple followup transformations, or because the pipeline is expanded and modified as data is explored. The shredded pipeline ensures scalability throughout the duration of the pipeline, removing the need to introduce intermediate grouping operations with the help of this succinct representation.

The shredded compilation employs the *shredding transformation*, which transforms programs that operate on nested data into a set of programs that operate on flat data; the resulting set of programs is the *shredded program*. Nested inputs are therefore required to be encoded as a set of flat relations; this is the *shredded input*. The shredded input and shredded program are provided as a succinct representation, where any attribute corresponding to a nested collection is referenced in the flat program using an identifier, known as a *label*. Labels encode necessary information to reassociate the levels of the shredded input. Reassociation is required when a specific level of the shredded program navigates over multiple levels of the shredded input, or when the output is returned as a nested type.

Figure 3 provides a high-level overview of the shredded compilation route, which produces a Spark application that defines the shredded program. The shredding transformation is hidden from the user and a user never interacts with shredded representations directly. Further details of the shredding transformation are described in [43].

Given the transformation to flat representation, the shredded compilation route supports distribution beyond top-level attributes. The succinct representation supports a more light-weight execution that replaces upper and lower-level attributes with labels; this results in reduced data transfer by means of shuffling and provides support for *localized operations*, which are operations that can be directly applied to the level specified in the input program. Shredding can be necessary for scaling for a small number of top-level objects and large/skewed inner collections [43]. Further performance benefits are presented in the Results section. We now continue with an explanation of shredding by example.

The shredded representation of `Occurrences` consists of three data sources:

- a top-level source of `Occurrences`, denoted `Occurrences_top` that returns data with a flat type

  [{ `sid` : *string*, `contig` : *string*, `start` : *int*, `end` : *int*, `reference` : *string*, `alternate` : *string*, `mutationId` : *string*, `candidates` : $Label_0$ }],

- the first-level source, denoted `Occurrences_candidates`, which has a flat datatype extending the type of `candidates` with a label attribute of *Label* type

  [{ `label` : $Label_0$, `gene` : *string*, `impact` : *real*, `sift` : *real*, `poly` : *real*, `consequences` : $Label_1$ }],

- and the second-level source which extends the type of `consequences` with a label attribute of *Label* type, denoted `Occurrences_candidates_consequences`

  [{ `label` : $Label_1$, `conseq` : *string* }].

The relationships between the shredded representations can be conceptualized as a database schema, with labels representing foreign-key dependencies. The `candidates` attribute in `Occurrences_top` is then a foreign key that references the primary key of `Occurrences_candidates` at `label`. Therefore, the reconstruction of nested output, known as *unshredding*, can be achieved by reassociating the shredded sources based on these relationships.

TraNCE then translates the nested program into a series of programs that operate on these flat inputs; i.e. constructs the shredded program. We now review the shredding transformation on the extended running example `TGHybridScores`.

Recall that the `TGHybridScores` program starts with the `dedup` operation that returns a collection of distinct tumor sites that will later be used for grouping. The first program returned from the shredding transformation is the shredded program `TumorSites_top`. The expression assigned to `TumorSites` operates over a flat input and returns flat output, so the shredding transformation essentially returns the identity:

```
TumorSites_top ⇐
 dedup(for s in Samples_top union
  [{tumorsite := s.tumorsite}])
```

The shredding transformation continues on the expression assigned to `TGHybridScores`, returning a series of three programs; collectively, the shredded `TGHybridScores` program. The first program represents the top-level collection, `TGHybridScores_top` with the `samples` attribute containing only a label reference.

```
TGHybridScores_top ⇐
for t in TumorSites_top union
  [{tumorsite := t.tumorsite,
    samples := NewLabel2({tumorsite := t.tumorsite})}]
```

The type of `TGHybridScores_top` is: [{ `tumorsite` : *string*, `samples` : $Label_2$ }]. There are no nested collection attributes, so this is indeed a flat collection. Further, the label of the `samples` attribute encodes only the necessary information to reconstruct the nested output, which in this case is the binding of `tumorsite`.

The program `TGHybridScores_samples` defines the succinct representation of the first-level expression, represented by the following program:

```
for s in Samples_top union
 [{label : NewLabel2({tumorsite := s.tumorsite}),
  tumorsite := s.tumorsite, sid := s.sid,
  scores := NewLabel3({sid := s.sid})}])
```

The type of `TGHybridScores_samples` is: [{ `label` : $Label_2$, `sid` : *string*, `scores` : $Label_3$ }].

The `label` expression defines a label that encodes the same information as the `samples` field in `TGHybridScores_top`. This is the same database-style representation seen with the shredded inputs. The `label` attribute is the primary key of `TGHybridScores_samples` and `TGHybridScores_top` references this with a foreign key at `samples`. The `scores` attribute encodes only the `sid` information that is needed in the next-level expression.

The program `TGHybridScores_samples_scores` defines the succinct representation of the lower-level expression, represented by the final program:

```
sumBy^{score}_{sid,gene}(
for o in Occurrences_top union
 for t in Occurrences_candidates union
  if o.candidates == t.label then
  for c in CopyNumber_top union
   if o.sid == c.sid && t.gene == c.gene then
    [{sid := NewLabel3({sid := o.sid}), gene := t.gene,
     score := t.impact * (c.cnum + 0.01) * t.sift * t.poly}])
```

The type of `TGHybridScores_samples_scores` is: [{ `label` : $Label_3$, `gene` : *string*, `score` : *real* }].

The `TGHybridScores_samples_scores` program navigates the top and first level of the shredded representation, using a conditional to reassociate these two shredded representations based on their label attributes. The shredded representation allows nested operations to work directly on the level assigned

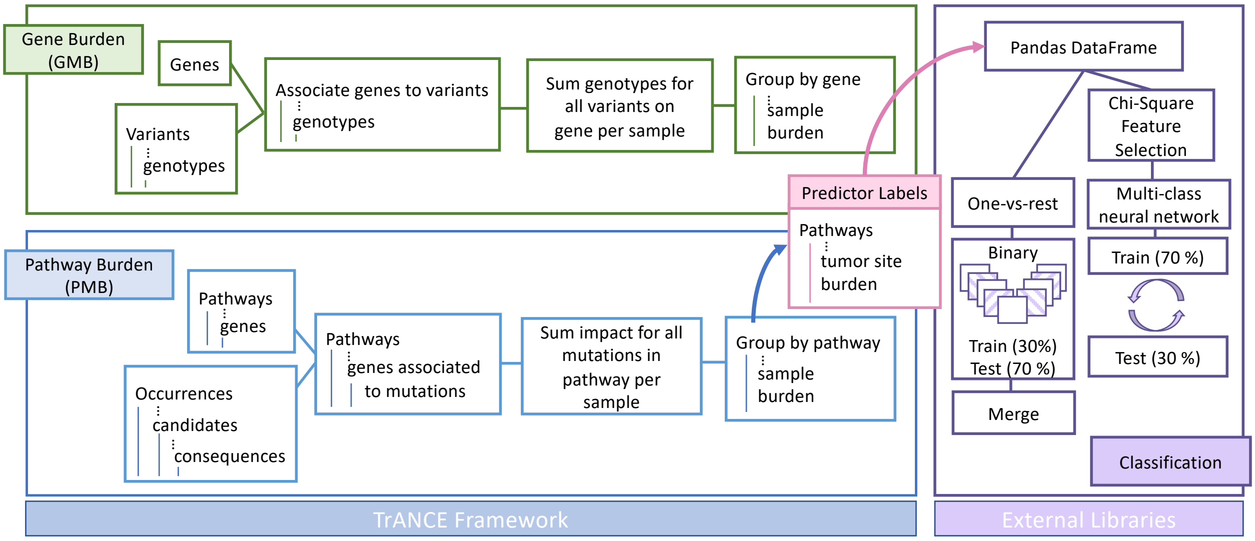

Figure 4. Workflow diagram representing the burden-based analyses for both genes and pathways, and downstream classification problem. The results of the pathway burden analysis feed into a classification analysis using multi-class and one-vs-rest methods to predict tumor of origin.

in the input program. This is an example of a localized operation that results in light-weight execution of nested data, removing the need to carry around redundant data.

The three programs associated to `TGHybridScores` are important for maintaining distribution of the nested values during program evaluation. The first program defines a collection of tumor site information; this avoids distributing the data based on a small number of top-level tuples. The second program defines a collection of sample information, further ensuring the distribution of the lowest level of nesting. The final program defines the bulk of the analysis, isolating the aggregation to the level where it is specified in the input program, and enabling execution of the aggregate without carrying around extra information from the parent.

Skew-Resilient Processing

Analytical pipelines often contain processes that associate items based on a shared attribute, such as the grouping by tumor site in tumor-grouped analysis. The execution of lines 6-7 of `TGHybridScores` will move all data belonging to a specific tumor site to the same partition - treating tumor site as a key. The TCGA-based `Occurrences` data source will contain significantly more samples for certain tumor sites than others; for example, there are 1100 patients associated to the breast cancer dataset (BRCA) and 51 patients associated to the lymphoma dataset (DLBC). The grouping operation will move all mutations associated to the 1100 BRCA patients to the same node and the whole of DLBC to another node. This will result in extreme imbalances of data across nodes leading to two main issues. First, the movement of a large amount of data to the same location could completely overwhelm the resources on that node - which is likely the case for pathway and gene family groupings. Second, any downstream computation of these groups will lead to significant bottlenecks in execution time; for instance, a simple count operation over the 444000 BRCA occurrences takes 32x that of the 8115 somatic occurrences of DLBC. Regardless of the specific operation, these distribution issues are a consequence of *skew*. Skew-related issues can easily burden an analysis and can be hard for high-level programmers to diagnose.

In distributed processing systems, skew is a consequence of key-based partitioning where all values with the same key are sent to the same partition; thus, skew is a problem even for flat datatypes. TraNCE automatically estimates skew-related bottlenecks at runtime and dynamically alters the query execution strategy to overcome skew. The core of the skew-handling procedure is the identification of *heavy keys*, which are keys with so many associated values that moving them all to the same partition would overwhelm the resources of that node. The framework uses a sampling procedure to identify heavy keys. There are four strategies available in the skew-handling procedure: full, partial, sample, and slice. Full is the most accurate method, fully identifying heavy keys based on all values across all partitions. Partial identifies heavy keys based on values locally within each partition. Sample identifies heavy keys by randomly sampling a subset (default 10%) of each partition. Since access to data within a partition is via an iterator interface, all of these methods require one full iteration over each partition, which can be expensive for large partitions. Slice evaluates heavy keys based on the first range of values (default 1000) in the partition. The sampling percent and slice range are all user-configurable. All methods categorize a key as heavy when the associated values make up a user-specified threshold of the total value (default 2.5%). All other keys are considered light.

Light keys follow the skew-unaware execution strategy. All heavy keys are subject to a broadcast-based execution strategy that prevents the movement of associated values to the same node. Broadcast is a feature of distributed processing platforms, which takes a set of values and duplicates them on each node. This means that the heavy values of one input are sent to the heavy values of another input, and the computation proceeds locally without shuffling any values. This means that values associated to heavy keys are not moved, which dramatically reduces the memory footprint of a task that would otherwise be memory intensive.

Both compilation routes leverage skew-handling methods that maintain proper distribution of values associated to heavy keys. Given that the shredded representation ensures distribution of inner-collections, the shredded compilation method is better-suited to deal with skew-related issues that arise from large nested collections and/or top-level distribution.

Code generation

The code generation stage translates a *TraNCE* program into a parallel data flow described in the Spark collection API, such as the application in Figure 2.

Input and output collections are modeled as Spark *Datasets*, which are strongly-typed, special instances of the native distributed collection type in Spark - Resilient Distributed Datasets (RDDs) [58]. Datasets are used because the alternative encoding – using RDDs of case classes – incurs much higher memory and processing overheads [41]. Datasets map to relational schemas and also allow users to explicitly state which attributes are used in each operation, providing valuable meta-information to the Spark optimizer.

Since nested inputs are represented as a collection of flat relations in the shredded pipeline, the shredded representation of data sources is merely a collection of

Spark Datasets. The tables representing the nested levels contain a label column (`label`) as key; these collections have a label-based partitioning guarantee, which is a key-based partitioning guarantee where all values associated to the same label reside on the same partition. Top-level collections that have not been altered by an operator have no partitioning guarantee and are distributed by the default, round-robin strategy.

The code generator can produce both Spark applications and Apache Zeppelin notebooks. Spark applications generate a single application file that can be executed via command-line. Notebooks can be imported into the Apache Zeppelin web-interface where users are able to further interact with the outputs of the generated code. Notebook generation was designed to provide initial support for users to interface with external libraries, such as pyspark [38], scikit-learn [39] [RRID:SCR_002577], and keras [26]. The notebooks rely on Zeppelin to translate Scala Datasets into Pandas DataFrames for easier interaction with machine learning and other advanced statistical packages. This is merely a first step towards integrating more advanced analytics in the system.

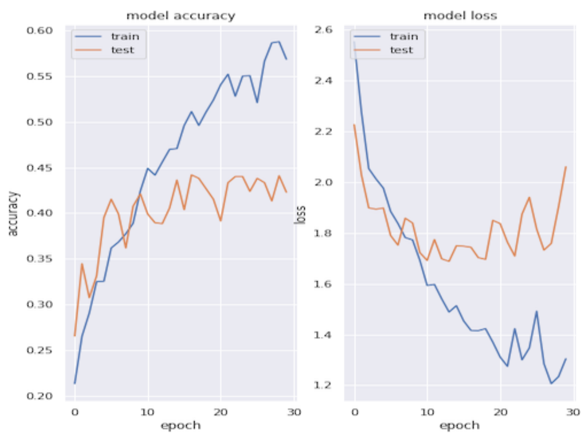

Figure 5. The accuracy and loss of the multi-class neural network for tumor tissue site.

Results

This section presents a collection of *TraNCE* programs and performance-related experiments that illustrate the different features of the platform. We first review two use cases that focus on research applications, then use the queries to measure system performance. The first use case is a single-omics analysis that builds mutational burden-based feature sets for use in external learning frameworks. The second use case is a multi-omics analysis pipeline that identifies driver genes in cancer, using nested input and constructing nested intermediate results to return flat output. We highlight the advantage of distributed computing for these use cases using a variety of cluster configurations and increasing data size. The third use case focuses on clinical applications and is designed to mimic requests a clinician could make from a user-interface that support multi-omics data integration. The final section presents an overview of how the shredded representation can leverage sharing.

Where relevant, we present the performance of the standard and shredded compilation routes. While previous results have shown that the standard compilation of *TraNCE* out performs several external competitors, including SparkSQL [41], the standard route is used as a representative of the flattening methods used in these systems and acts as a baseline for the scalable shredded compilation route. All experiments are run using Spark 2.4.2, Scala 2.12, Hadoop 2.7. Runtimes are measured after caching all inputs into memory. The schemas of each of the query inputs are described in the supplementary material. Performance-related results of high importance are highlighted in the relevant sections.

Application 1: Mutational Burden

High mutational burden can be used as a confidence biomarker for cancer therapy [12, 7]. One key measure is *tumor mutational burden* (TMB), the total number of somatic mutations present in a tumor sample. Here we focus on two subcalculations of TMB: gene mutational burden (GMB) and pathway mutational burden (PMB). GMB is the total number of somatic mutations present in a given gene per tumor sample. PMB is the total number of somatic mutations present in a given pathway per tumor sample. These burden-based analyses provide a basic measurement of how impacted a given gene or pathway is with somatic mutations. Mutational burden can be used directly as a likelihood measurement for immunotherapy response [12], or can be used as features for a classification problem.

The progression of some cancers could make it impossible for a clinician to identify the tumor of origin [24]. The ability to classify tumor of origin from a cohort of cancer types can be clinically actionable, providing insights into the diagnosis and type of treatment the patient should receive. For the burden-based use case, we aim to predict tumor of origin from a pancancer dataset.

Figure 4 summarizes the burden-based analyses that calculate GMB and PMB and then perform downstream classification to predict tumor of origin. Each analysis starts by assigning the mutations of each sample, from either `Variants` or `Occurrences`, to the respective gene or pathway. Once assigned, the results are aggregated to return total mutation counts for each gene or pathway producing GMB or PMB values for each sample. The result of the PMB analysis is annotated with tumor site predictor labels from `Samples`, and converted to a Pandas DataFrame to perform two multi-classification methods to predict tumor of origin from a pancancer dataset. We now present the TraNCE programs for these analyses and describe the downstream learning application.

Given that a pathway is represented as a set of genes, GMB is a partial aggregate of pathway burden; i.e. PMB is the sum of all the gene burdens for each gene belonging to a pathway. We thus show the gene burden program using mutations from `Variants` and the pathway burden program using somatic mutations from `Occurrences`.

The `Variants` data source is based the VariantContext [21] object, used to represent variants from a Variant Call Format (VCF) file. This data structure represents one line, i.e. one variant, from a VCF file. Variants are identified by chromosome, position, reference and alternate alleles, and associated genotype information for every sample. We use an integer-based categorical assignment to genotype calls to support analyses; 0 is homozygous reference with no mutated alleles, 1 is heterozygous with 1 mutated allele, and 2 is homozygous alternate with 2 mutated alleles. The type of `Variants` is:

$$[\,\{\,\texttt{contig} : \textit{string}, \texttt{start} : \textit{int}, \texttt{reference} : \textit{string}, \texttt{alternate} : \textit{string}, \texttt{genotypes} : [\,\{\,\texttt{sid} : \textit{string}, \texttt{call} : \textit{int}\,\}\,]\,\}\,].$$

Gene burden. The gene burden program performs a VCF-based analysis using the `Variants` data source. The program first iterates `Genes` creating a top-level gene group, and then performs a sum-aggregate of the nested genotype calls for each sample corresponding to that gene. Variants are associated to a gene if it lies within the mapped position on the genome.

```
GMB ⇐
for g in Genes union
 [{gene := g.gene, burdens :=
  sumBy^burden_sid(
   for v in Variants union
    if v.contig == g.contig && v.start >= g.start
     && v.end <= g.end then
      for c in v.genotypes union
       [{sid := c.sid, burden := c.call}])}]
```

The output type is:

$$[\,\{\,\texttt{gene} : \textit{string}, \texttt{burdens} : [\,\{\,\texttt{sid} : \textit{string}, \texttt{burden} : \textit{real}\,\}\,]\,\}\,].$$

The `GMB` program could be altered to include a larger flanking region by changing the equalities on `start` and `end` to use a range.

Pathway burden. The `PMB` program uses the annotations within the `Occurrences` data source to determine gene association. These burden scores are measured within a wider scope than the `GMB` program. When a candidate gene set is created based on a large flanking region, the pathway burdens could

be dramatically over-estimated. To account for this, the program uses impact information instead of the number of alleles to measure the mutational burden of a pathway.

```
PMB ⇐
for p in Pathway union
  [{pathway := p.pathway, burdens :=
    sumBy_sid^score(
      for o in Occurrences union
        for t in o.candidates union
          for g in p.genes union
            if g.gene == t.gene then
              [{sid := o.sid, burden := t.impact}])}]
```

The output type is:

$$[\,\{\,\texttt{pathway} : \textit{string}, \texttt{burdens} : [\,\{\,\texttt{sid} : \textit{string}, \texttt{burden} : \textit{real}\,\}\,]\,\}\,].$$

A simple version of the PMB program could use raw counts, which we will use for downstream classification analysis. A more complex version could combine multiple impact attributes, such as impact, poly, and sift, to provide a better estimate of burden.

Classification with burden-based features. We now consider how the burden-based programs can be employed to create feature vectors for a learning classifier. Classification of tumor origin has been previously explored with various cancer biomarkers [29, 62, 54, 25]. The goal of our classification problem is to identify tissue of origin from the whole TCGA dataset using pathway burden features based on raw mutation count.

The classification process starts by preparing the PMB output for classification, labeling each pathway burden feature with the associated label:

```
for p in PMB union
 [{pathway := p.pathway, burdens :=
   for b in p.burdens union
     for s in Samples union
      if b.sid == s.sid then
       [{tumorsite := s.tumorsite, burden := b.burden}]}]
```

In order to interface with external machine learning libraries, the burden-based programs are compiled into Zeppelin notebooks where the output is available once the program is executed. Learning procedures can then be applied directly in Spark/Scala, or the ZeppelinContext can be used to read a Spark DataFrame as a Pandas DataFrame. For this example, we focus on the Pandas representation to highlight how a user can interact with TraNCE outputs using their external library of choice. Given this data processing pipeline, TraNCE is used for the heavy lifting portion that integrates and restructures the datasets to produce feature matrices; the assumption being that the data is reduced to a size reasonable enough for the in-memory processing of standard statistical libraries.

Once represented as a Pandas DataFrame, the data is split for training and testing using scikit-learn and neural networks are constructed with keras. For the whole of the TCGA dataset, we use a minimum cut-off of 200 representative samples. This leaves 9 different tumor tissue sites available for classification: breast, central nervous system, colon, endometrial, head and neck, kidney, lung, ovary, and stomach.

We first train a fully-connected, feed-forward multi-class neural network for tumor tissue site, using 1600 pathways selected by the Chi-squared test as the features. The neural network uses LeakyReLu [57] with *alpha = 0.05* as the activation function, and we utilize dropout layers [46] with *dropout_rate = 0.3* after each fully connected layer (dense layer) before the output. This model is trained using a categorical cross-entropy loss function and an Adam optimizer [28]. The network has a Softmax output, which can be interpreted as a probability distribution over 9 different tumor tissue sites. The data is randomly split into two folds, 70% for training and 30% for testing.

Next, we extend the previous method via the "one-vs-rest" method [61], which decomposes a multi-classification problem into multiple binary classification problems and each binary classifier is trained independently. For every sample, only the most "confident" model is selected to make the prediction.

Each binary classifier is a fully connected, feed-forward neural network, using all 2230 pathways as the features. These are set up the same as the multi-class networks, except with dropout layer *dropout_rate = 0.15* and a binary cross-entropy loss function. The binary networks have Sigmoid output, which can be interpreted as a probability of a certain type of tumor tissue site corresponding to this model. For each model, the data is randomly split into two folds as with the tumor-site network.

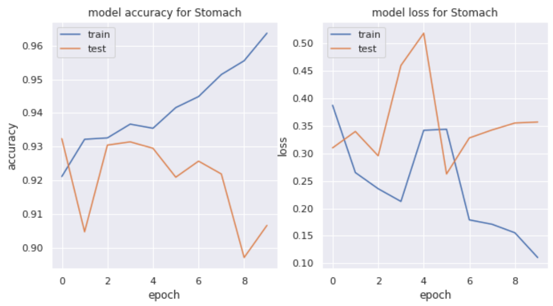

(a) Stomach

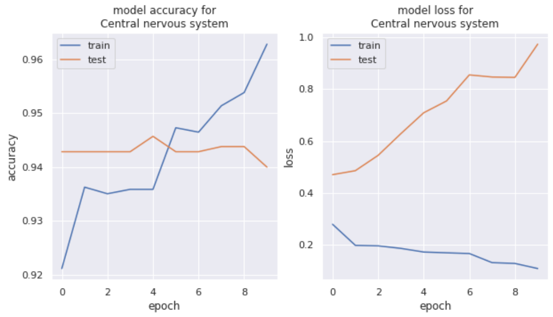

(b) Central nervous system

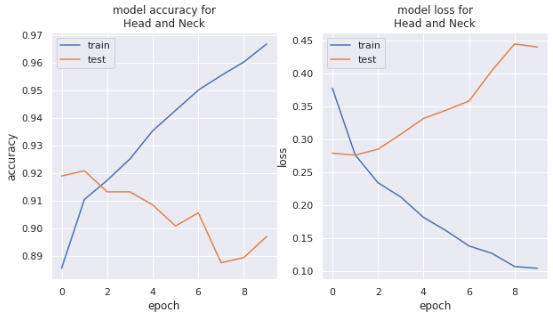

(c) Head and neck

Figure 6. Accuracy and loss for the tumor tissue site based binary network, includes results for the three worst-performing classes from the multi-class network.

We train 9 independent binary classifiers for each type of tumor tissue site. These binary models predict the likelihood that the given pathway burden measurements of a patient are associated with the tumor site represented by that model. After training each binary model, predictions are made using the entire dataset, and the computed results are merged. The probabilities from all models are compared for each patient from the testing dataset, classifying the patient according to the highest likelihood. For example, suppose we have two models, a breast model that predicts a breast-site likelihood of 0.8 and a lung model that predicts a lung-site likelihood of 0.6 for the same patient. The system compares these two probabilities and classifies tumor of origin as breast.

Difference in sampling procedures aside, the multi-classifier and the binary models in the one-vs-rest method have one key difference. When using pathway burden features, pathways that are highly correlated with a specific tumor site could be overpowered by pathways that show strong signal for cancer in general. The multi-classifier could compromise features specific to tumor of origin in an attempt to achieve best performance overall. This can lead to particularly

inaccurate results when the data distribution is uneven. The binary models are eager to select the best feature weights for the representative tumor of origin, providing more opportunities for tumor-specific features to stand out.

Multi-classification results. Figure 5 shows the accuracy and loss of the multi-class neural network for tumor tissue site for 30 epochs. The overall accuracy is 42.32%, calculated from the confusion matrix adding all 444 correctly predicted labels together and dividing by the 1049 testing samples. Most misclassifications were predicted to be breast cancer, likely attributed to the data imbalance problem of the training dataset. An imbalanced data distribution forces a model to learn features corresponding to highly populated labels, reducing training loss while skewing overall prediction performance.

Different types of cancer may not contain enough dominant features for a simple multi-class model to distinguish differences among tumor origin site. Even pathways that play a key role in any cancer, such as pathways specific to disruption in cell-cycle, could not be providing enough signal to act as a determinant for cancer types. This could be because other pathways are washing out the signal of more important pathways, or it could simply mean that pathway burden alone is not providing the whole story. Thus, future multi-class problems in this domain should consider integrating other features, such as additional genomic measurements, or filter pathways based on prior knowledge of the cancer types in question.

One-vs-rest classification results. Figure 6 displays the accuracy and loss of three binary networks for 10 epochs. We present the three worst-performing classes from the multi-class network: stomach, head and neck, and central nervous system, which all resulted in testing accuracies above 90% in the one-vs-rest method. The accuracy and loss of the other binary models are provided in the supplementary material. The combined accuracy of all binary models is 78.44%, calculated as the correctly predicted labels (2744) divided by total samples (3498). Overall performance of the one-vs-rest method is far better than the multi-classifier performance.

Further exploration into the pathway signal profiles of each tumor site could be considered for future work. Gene burden performance could be compared to the performance of pathway burden in order to identify genes that are the main drivers for pathway signal. The identification of predominant pathways and genes for certain tumor sites could provide insight into specific cancer profiles and determine the overall confidence of using burden-based features for tumor site classification.

The burden-based use case exemplifies how TraNCE can handle data integration tasks, and more specifically, integration tasks that produce feature vectors for classification problems. In addition, this use case shows how users can interact with popular learning packages within a notebook environment without the overhead associated with manually integrating data sources.

Application 2: Multi-omics cancer driver gene analysis

Mutations that play a driving role in cancer often occur at low frequency [17], making cohort analysis across many samples important in their identification. Further, a cancer profile is more than just a consequence of a single mutation on a single gene. Gene interactions, the number of such genes, and their expression levels can provide a more thorough look at cancer progression [8]. This use case focuses on such a multi-omics analysis, which defines a set of programs that integrate annotated somatic mutation information (`Occurrences`), copy number variation (`CopyNumber`), protein-protein network (`Network`), and gene expression (`GeneExpression`) data to identify driver genes in cancer [60]. This analysis provides an integrated look at the impact cancer has on the underlying biological system and takes into account the effects a mutation has on a gene, the accumulation of genes with respect to both copy number and expression, and the interaction of genes within the system. The programs of the driver gene analysis work in pipeline fashion, where the materialized output from one program is used as input to another later on in the pipeline.

Figure 7 provides an overview of the cancer driver gene analysis. The pipeline starts with the integration of mutation and copy number variation to produce a set of hybrid scores for each sample. The hybrid scores are then combined with protein-protein network interactions to determine effect scores. The effect scores are further combined with gene expression information to determine the connection scores for each sample. The analysis concludes by combining the connection scores across all samples, returning connectivity scores for each gene. The genes with the highest connectivity scores are considered drivers. We now detail each of the steps and conclude with some performance metrics using the two compilation routes.

Hybrid scores. The hybrid score program `HybridScores` is the first step in the pipeline, and is an advanced version of the `SGHybridScores`. The program below describes the process of creating hybrid scores based on the `Occurrences` input. Here, `Samples` provides a map between `sid` and `aliquot` used to join `CopyNumber`, and the hybrid scores are then determined for every `aliquot`. In addition, conditionals are used to assign qualitative scores based on the human-interpretable level of impact (`impact`). The `SOImpact` information is used to integrate values from the nested `consequences` collection into the hybrid score.

```
HybridScores ⇐
for s in Samples union
 [{sid := s.sid, aliquot := s.aliquot, scores :=
  sumBy^{score}_{gene}(
   for o in Occurrences union
    if o.sid == s.sid then
     for t in o.transcripts union
      for n in CopyNumber union
       if s.aliquot == n.aliquot &&
          n.gene == t.gene then
        for c in t.consequences union
         for v in SOImpact union
          if c.conseq == v.conseq then
           [{gene := t.gene, ... score := ...
            let impact :=
             if t.impact == "HIGH" then 0.8
             else if t.impact == "MODERATE" then 0.5
             else if t.impact == "LOW" then 0.3
             else if t.impact == "MODIFIER" then 0.15
             else 0.01
           in impact * v.value * (n.cnum + .01) * sift * poly}]
    )}]
```

The output type of `HybridScores` is:

$$[\{\texttt{sid} : \textit{string}, \texttt{aliquot} : \textit{string}, \texttt{scores} : [\{ \texttt{gene} : \textit{string}, \texttt{score} : \textit{real}\}]\}].$$

The `HybridScores` program must persist the `aliquot` attribute in order to associate more genomic measurements related to that aliquot later in the pipeline. These hybrid scores now provide a likelihood score of a gene being a driver within a specific aliquot based on both accumulated impact of somatic mutations and copy number variation. The analysis continues to integrate further information to increase the confidence of driver gene scores.

By Sample Network. The second step in the pipeline `HybridNetworks` builds individual aggregated networks for each (`sid`, `aliquot`) pair in the materialized output of `HybridScores`. For each sample, we take the product of the `score` and edge protein `distance` for each edge in the network; genes are associated to proteins based on the mapping provided in the `Biomart` gene map table. The sum aggregate of these values is then taken for each node protein in `Network`, while maintaining top-level sample groups.

```
HybridNetworks ⇐
for h in HybridScores union
 [{sid := h.sid, aliquot := h.aliquot, nodes :=
  sumBy^{score}_{gene}(
   for g in h.scores union
    for b in Biomart union
     if g.gene == b.gene then
      for n in Network union
       for e in n.edges union
        if e.edgeProtein == b.protein then
         [{nodeProtein := n.nodeProtein,
            score := e.distance * h.score}])}]
```

The output type of this query is:

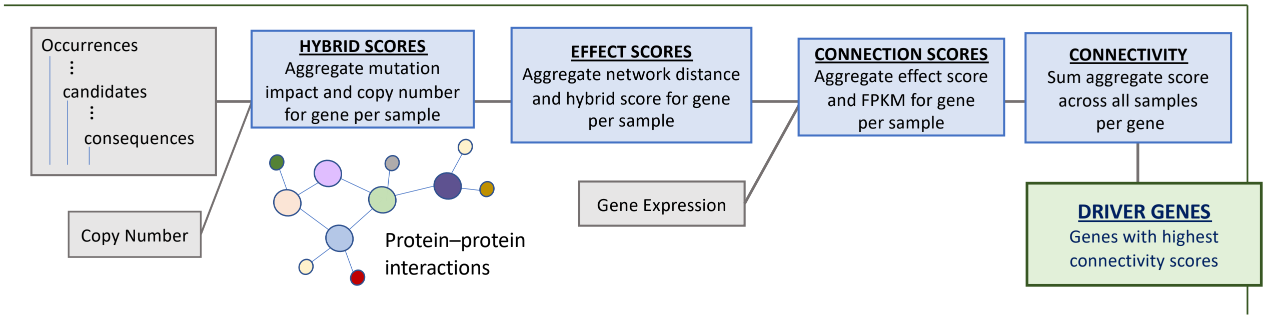

Figure 7. Summary of the cancer driver gene analysis. The pipeline starts by integrating somatic mutations and copy number variation, further integrates network information, and gene expression data. The genes with the highest connectivity scores are taken to be drivers.

$$[\,\{\,\texttt{sid} : \textit{string}, \texttt{aliquot} : \textit{string}, \texttt{nodes} : [\,\{\, \texttt{nodeProtein} : \textit{string}, \texttt{score} : \textit{real}\,\}\,]\,\}\,].$$

The `HybridNetworks` program produces an intermediate score for each protein in the network by weighting the hybrid scores of nearby proteins in the network (edges) based on their distance scores; thus, this is an intermediate aggregation of the network data with the hybrid scores using only the edges in the network.

Effect scores. To complete the integration of network data with the hybrid scores, the next step is to integrate the nodes in the `Network` to produce effect scores. Effect scores are produced by combining the accumulated edge-based hybrid scores from `HybridNetworks` with the hybrid score for each protein node for each sample in the materialized output of `HybridScores`. As in `HybridNetworks`, genes are associated to proteins using the `Biomart` mapping table.

```
EffectScores ⇐
for h in HybridScores union
 [{sid := h.sid, aliquot := h.aliquot, scores :=
  for s in HybridNetworks union
   if h.sid == s.sid && h.aliquot == s.aliquot then
    for n in s.nodes union
     for b in Biomart union
      if n.nodeProtein == b.protein then
       for y in h.scores union
        if y.gene == b.gene then
         [{gene := y.gene, score := n.score ∗ h.score}]}]
```

The output type of this query is:

$$[\,\{\,\texttt{sid} : \textit{string}, \texttt{aliquot} : \textit{string}, \texttt{scores} : [\,\{\, \texttt{gene} : \textit{string}, \texttt{score} : \textit{real}\,\}\,]\,\}\,].$$

At this point, the effect score is another likelihood measurement for a gene being a driver gene for cancer. The analysis now continues to add confidence to the effect score by further integrating gene-based measurements.

Connection scores. The `ConnectScores` program calculates the connection scores. A connection score is the product of the effect score and the FPKM value from the `GeneExpression` table. Gene expression data is combined with the materialized output of `EffectScores` to determine the connection scores for each gene within every sample.

```
ConnectScores ⇐
for e in EffectScores union
 [{sid := e.sid, aliquot := e.aliquot, scores :=
  sumBy^score_gene(
   for e in s.scores union
    for g in GeneExpression union
     if e.gene == g.gene then
      [{gene := e.gene, score := e.score ∗ g.fpkm}])}]
```

The output type of this query is:

$$[\,\{\,\texttt{sid} : \textit{string}, \texttt{aliquot} : \textit{string}, \texttt{scores} : [\,\{\, \texttt{gene} : \textit{string}, \texttt{score} : \textit{real}\,\}\,]\,\}\,].$$

Given the pipeline nature of these queries, the connection scores for each gene are the accumulated somatic mutation, copy number, protein-protein network, and gene expression data for each sample. The connect score can be used to determine the likelihood of a gene being a driver in a specific sample. In theory, this likelihood measurement should have more confidence than the hybrid or effect scores.

Gene connectivity. At this point in the analysis, all the genomic measurements have been integrated to produce high-confidence likelihood connection scores for each gene within each sample. The final step is to combine across all samples to identify the highest scoring genes over all samples; this is the gene connectivity. Gene connectivity uses the materialized output of `ConnectScores`, summing up the connection scores for each gene across all samples. The genes with the highest connection scores are taken to be drivers.

```
Connectivity ⇐
sumBy^score_gene(
for s in ConnectScores union
 for c in s.scores union
  [{gene := c.gene, score := s.score}])
```

The output type is:

$$[\,\{\,\texttt{gene} : \textit{string}, \texttt{score} : \textit{real}\,\}\,]$$

Collectively, these five programs make up the cancer driver gene analysis. The final output of of `Connectivity` is sorted and the top genes are investigated as likely driver genes for cancer. Further confidence can be gained by fine-mapping techniques [27].

This analysis follows the workflow of [60], which terminates with an identification of driver genes. The three top driver genes reported from our analysis were TP53, FLNA, and CSDE1. All these genes have previously been reported as important for their role in cancer. Future work should explore the pancancer results of this analysis, potentially comparing tumor-site specific driver genes to the identified pancancer driver genes. Naturally, we could also use some of the intermediate scores as features for learning algorithms, as with the previous case study.

The runtime performance of this pipeline was presented in [43], showing that the shredded compilation route was the key to scalability for larger datasets. We use the queries of this analysis to demonstrate the scalability of the framework in the context of biology in the next section.

Scalability experiments

This section uses the above two use cases to illustrate the scalability benefits of the platform for biological pipelines. We use the burden-based analysis to show scalability of the shredded compilation route for increasing data size and constant cluster size, using the standard compilation method as a baseline. We then use the driver gene analysis to measure scalability for constant data size

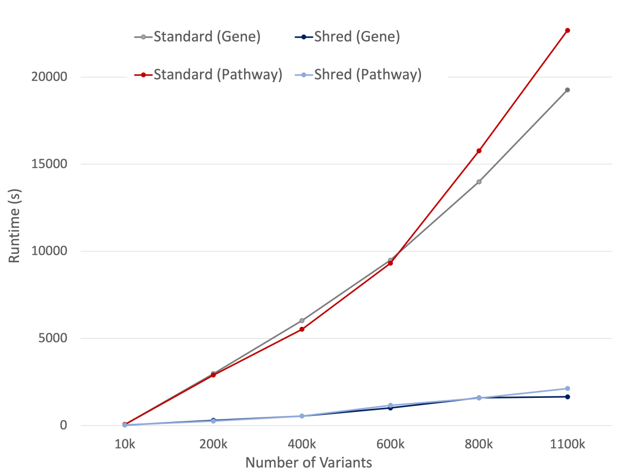

Figure 8. Performance comparison between the standard and shredded pipeline on gene and pathway burden analysis using the 1000 Genomes dataset.

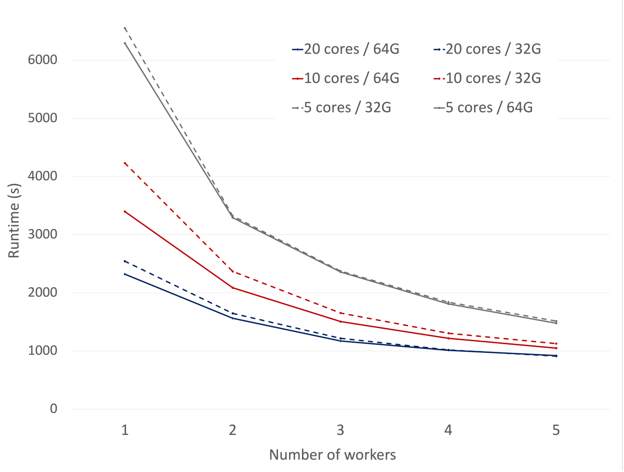

Figure 9. Scalability for the driver gene analysis measured using `HybridScores` and `HybridNetworks` programs for a variety of cluster configurations.

and increasing cluster size. These experiments highlight the scalability of the shredded compilation route.

Increasing data size. We use the burden-based analysis to show the performance of the shredded compilation route for an increasing number of top-level records. We run Spark with one worker, 10 executors, 2 cores and 20 Gigabyte (GB) memory per executor, and 16GB of driver memory. Due to resource limitations of this cluster, we use the publicly available chromosome 22 from phase 3 of the 1000 Genomes Project [4, 49]; this is a 11.2GB dataset representing 2504 samples.

Figure 8 displays the runtimes of the standard and shredded compilation for the gene burden and pathway burden analysis for an increasing number of variants from the VCF-based `Variants` data source. The results show that flattening methods of the standard route are quickly overwhelmed as the number of variants increase; whereas, the shredded route increases at a much slower rate. In addition, after 600-thousand variants the standard pathway burden run increases at a greater rate than the corresponding gene burden run. The shredded method exhibits two main advantages. First, the succinct representation avoids carrying around extra data, such as the genotypes information when `Variants` are joined with `Genes`. Second, the result of flattening `Variants` will have a large amount of items. The whole file contains roughly 1103600 variants and more than 2500 samples, which produces a result with over 2.7 billion items. The performance benefits exhibited in this experiment are only for a single chromsome; as such, distributed computing becomes even more of a necessity when processing whole genomes or considering more samples. *Overall, these results highlight the advantage of the shredded representation even for the shallow nesting of the VariantContext structure.*

Increasing cluster size. This experiment uses queries from the driver gene analysis to assess scalability of the shredded compilation route as the amount of compute resources increase. Figure 9 displays the combined runtime for the first two, most expensive steps of the driver gene analysis - `HybridScores` and `HybridNetworks`- for an increasing amount of workers and a variety of cluster configurations. The queries are run with the pancancer datasets, including 280GB of `Occurrences` [23, 32], 4GB of `Network` [50], and 34GB of `CopyNumber` (34GB). We focus only on the shredded compilation route, since the standard compilation route was unable to perform for this scale of data.

We use an increasing number of cores (5, 10, 20) per worker to evaluate performance based on the number of processing units available to each worker. For each of these runs we provide 32G and 64G per worker to assess how available memory impacts performance. For each run workers are added with constant amounts of resources; for example, for the 5 cores and 32G run, the one worker mark represents 5 cores and 32G total, whereas the two worker mark represents 10 cores and 64G total. The number of cores and number of workers are used collectively to measure overall distribution.

The single worker runs exhibit the largest variation in runtime. While the single worker with 10 cores completes 14 minutes faster when more memory is available, the runtimes for the single workers with 5 and 20 cores are less impacted when memory increases. In general, the total number of cores and the amount of memory available to each working unit is more important when there are fewer workers available to a cluster.

The results show that all runs converge to a point where the shuffling overhead dominates the total execution time and what is left is not parallelizable. This shuffling overhead is related to the expense of the `HybridNetworks` program, which requires a significant amount of shuffling to perform a join on nested attributes. Our previous results reported 470G of shuffled data for the shredded compilation route; whereas the standard compilation crashed after shuffling nearly 2.1TB [43]. Even in a situation where large amounts of shuffling can not be avoided, the addition of five workers has saved 24 - 78 minutes depending on the amount of resources available to each worker. *Overall, these scalability results highlight how adding cluster resources will improve the performance of current data pipelines.*

Skew-handling experiments

In the skew-resilient processing section, we introduce an initial example based on grouping that describes the prevalence of skew in biomedical analyses. This experiment extends upon that example to show the benefits of using the skew-handling feature of TraNCE. We explore the cost of grouping `Occurrences` by tumor site, gene, pathway, and gene family. We use the 92 tumor sites represented in the TCGA dataset, 58-thousand genes from a gene map file, 2230 pathways based on curated gene sets from The Molecular Signatures Database (MSigDB) [33, 47], and the 8 gene family classifications also from MSigDB. Grouping by tumor site follows the structure of the extended running example `TGHybridScores`. The grouping of mutations by gene and pathway are represented by the `GMB` and `PMB` programs from Application 1. Gene families are a special instance of pathway, so this grouping also follows `PMB`.

We use a subset of the `Occurrences` dataset (12.3G), which represents 10% of the full dataset. Each program was run on a cluster with 5 workers each with 20 cores and 16G. Figure 10 displays the runtimes for the full, partial, sample, and slice skew-handling techniques as well as without skew-handling techniques (skew-unaware) for both the standard and shredded compilation routes. As in the previous experiments, the standard route is included to represent baseline flattening methods.

The tumor site and gene groupings exhibit low amounts of skew; all methods are able to run to completion with the skew-aware techniques only adding a slight overhead related to the cost of performing the heavy key calculation. While partitions can still be of disproportionate size for low amounts of skew, the amounts exhibited for these groupings are not enough to overwhelm any resources on a single node. In addition, the shredded runs benefit from the

natural skew-resilience of the shredded representation, which stores the groups as a Dataset instead of a nested collection. This overhead is exhibited in the standard compilation route runs; there is a 2.5x advantage to shredding when grouping by tumor site and an 80x advantage when grouping by gene. The standard compilation route was unable to perform at all for higher amounts of skew regardless of skew-handling techniques.

The pathway run exhibits moderate amounts of skew. Here the skew-unaware method is unable to perform at all, spilling 540G of data to disk before crashing; this is expected with increased amounts of skew since the values associated with heavy keys will completely overwhelm the resources on each node. The gene family run exhibits high amounts of skew. Again, the skew-unaware method is unable to perform at all. The skew-aware methods for pathway and gene family highlight the significant performance gains of of avoiding the key-based partitioning strategy for heavy keys. The performance gain increases for higher amounts of skew since the majority of the values will be associated to heavy keys and these values will remain stationary during the skew-aware operation.

The slice procedure exhibits interesting behavior for varying amounts of skew and the different compilation routes, highlighting benefits of this procedure for estimating heavy keys in large intermediates. For example, the slice procedure is the most expensive when skew is low and the shredded representation is used, whereas slice is the best performing for the corresponding standard route. In the standard procedure, `Occurrences` is flattened before joining with the gene table that will instantiate the nested mutation groups. This flattening produces more values for the skew-handling strategy to process, which is where the slice procedure performs better. On the other hand, the shredded representation runs the estimate on the lighter-weight, first-level source of `Occurrences` and ends up over estimating the heavy keys. Overestimating heavy keys can lead to longer processing times due to broadcasting larger intermediates.

The slice method performs best when using the shredded representation for the moderate and high amounts of skew in the pathway and gene family groupings. For these queries, full, partial, and sample have longer processing times due to over estimation. The slice procedure is the best performing of all methods, which can quickly estimate heavy keys while keeping the amount of broadcasted data low. Given that the full procedure should accurately identify all heavy keys, this suggests that it is advantageous to only apply skew-aware operations to the most immediately identifiable heavy keys than it is to fully estimate heavy keys. Our previous results had only shown benefits of the sample procedure using synthetic data. *These results show that when applied to real-world, biological datasets the slice procedure tends to have better performance overall.*

Application 3: Clinical exploratory queries

We now present a clinically-focused use case that highlights additional advantages of TraNCE. The identification of personalized diagnosis and treatment options is dependent on insights drawn from large-scale, multi-modal analysis of biomedical datasets. Practical clinical application of such targeted analyses require interfacing with electronic health record (EHR) systems, to provide a data processing environment that supports ease of integrating genomic, clinical, and other biomedical data linked to patients. For example, the Informatics for Integrating Biology and Beside (i2b2) [22] framework facilitates web-based cohort exploration, supporting selection and report generation on clinical attributes. Several proposed solutions for integrating genomic data into i2b2 have been proposed [14, 34, 45]. In these systems, genomic and clinical data are stored in separate databases and then combined in a backend plugin using the i2b2 API.

Figure 11 presents a schematic of an i2b2 instance that supports aggregate analysis with clinical and genomics data sources, i.e. `Occurrences` and `CopyNumber`. The programs of this use case are inspired by such a situation. A user makes a request from a clinical interface. This request represents an analysis that is sent to the backend. The backend application communicates to each of the external data sources to retrieve the necessary data and import them into a Spark processing environment. The application sends the computed results back to the user interface for viewing.

Each of the programs below compromise an analysis that would be performed by the backend application using *TraNCE*. A major difference from the prior

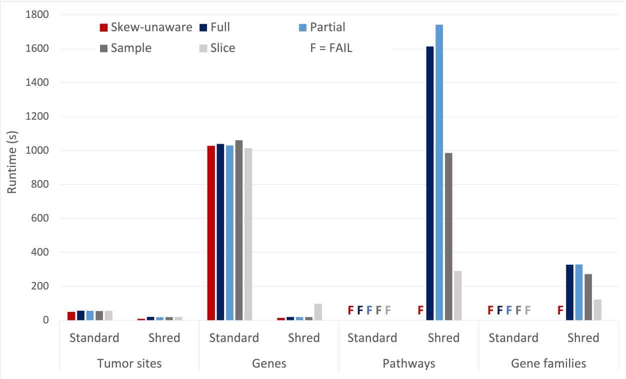

Figure 10. Performance comparison of the skew-handling techniques for both the standard and shredded compilation routes. Queries are organized based on increasing amounts of skew, such that tumor sites is representative of low skew and gene families of high skew.

use cases is that here we are not computing just flat aggregates. We are returning nested that will be explored interactively at the web interface. The output will reflect situations where the majority of data fields are returned for exploration by the user,. We now review three such applications, which perform a combination of restructuring, integration, and aggregation of `Occurrences`, `CopyNumber`, and `Samples`.

Group occurrences by sample. The `OccurGrouped` program groups the somatic mutation occurrences in `Occurrences` by sample based on `Samples`, producing a collection of nested mutation information for each sample. The program also associates a quantitative value to the consequences at the lowest level in the process, as seen previously in the `HybridScores` program from the driver gene analysis.

```
OccurGrouped ⇐
for s in Samples union
 [{sid := s.sid, mutations :=
  for o in Occurrences union
   if s.sid == o.sid then
    [{mutationId := o.mutationId, ..., candidates :=
     for t in o.candidates union
      [{gene := t.gene, ..., consequences :=
       for c in t.consequences union
        for i in SOImpact union
         if c.conseq == i.conseq then
          [{conseq := i.conseq, score := i.value}]}]}]}]
```

The ellipses represent all the additional fields from `Occurrences`. The output type of `OccurGrouped` is:

[{ `sid` : *string*, `mutations` : [{ `contig` : *string*, `start` : *int*, `end` : *int*, `reference` : *string*, `alternate` : *string*, `mutationId` : *string*, . . . , `candidates` : [{ `gene` : *string*, `impact` : *string*, `sift` : *real*, `poly` : *real*, . . . , `consequences` : [{ `conseq` : *string*, `score` : *real* }] }] }] }].

The `OccurGrouped` program groups a mutation data source, like `Occurrences`, based on sample. All information associated to a mutation is returned, with most of the fields of `Occurrences` persisted in the output. The result of this program could feed into a web-interface that provided a detailed view of annotated mutations across a cohort of patients.

Integrate copy number and occurrences, group by sample. The next program extends `OccurGrouped` by associating copy number information (`CopyNumber`) to each of the genes in the `candidates` collection for each mutation in `Occurrences`. The results are returned group by sample, and the majority of the fields from `Occurrences` are persisted in the output.

```
OccurCNVJoin ⇐
for s in Samples union
 [{sid := s.sid, mutations :=
  for o in Occurrences union
   if s.sid == o.sid then
```

```
[{mutationId := o.mutationId, ..., candidates :=
 for t in o.candidates union
  for g in CopyNumber union
   if g.gene == t.gene && g.sid == o.sid then
    [{gene := t.gene, cnum := g.cnum, ...,
      consequences :=
       for c in t.consequences union
        for i in SOImpact union
         if c.conseq == i.conseq then
          [{conseq := i.conseq, score := i.value}]}]}]}]
```

The output type of `OccurCNVJoin` is:

[{ `sid` : *string*, `mutations` : [{ `contig` : *string*, `start` : *int*, `end` : *int*,
`reference` : *string*, `alternate` : *string*, `mutationId` : *string*, . . . ,
`candidates` : [{ `gene` : *string*, `impact` : *string*,
`sift` : *real*, `poly` : *real*, `cnum` : *int*, . . . , `consequences` :
[{ `conseq` : *string*, `score` : *real* }] }] }] }].

This program exhibits the integration of copy number data on a nested attribute, without any aggregation. The `OccurCNVJoin` program addresses the situation where additional biomedical datasets are integrated for exploration in a consolidated view.

Aggregate copy number and occurrences, group by sample. The final clinical program combines all aspects of the first two programs and adds an additional aggregation. As in `OccurCNVJoin`, mutations are associated to copy number data to create an aggregate value with mutational impact from the nested `consequences` collection of `Occurrences`. The scores are returned for each candidate gene within each mutation, and the final output is grouped by sample.

```
OccurCNVAgg ⇐
for s in Samples union
 [{sid := s.sid, mutations :=
  for o in Occurrences union
   if s.sid == o.sid then
    [{mutationId := o.mutationId, ..., candidates :=
     sumBy^score_gene(
      for t in o.candidates union
       for g in CopyNumber union
        if g.gene == t.gene && g.sid == o.sid then
         for c in t.consequences union
          for i in SOImpact union
           if c.conseq == i.conseq then
            [{gene := t.gene,
              score := t.impact * g.cnum * i.value}])}]}]
```

The output type of `OccurCNVAgg` is:

[{ `sid` : *string*, `mutations` : [{ `contig` : *string*, `start` : *int*, `end` : *int*,
`reference` : *string*, `alternate` : *string*, `mutationId` : *string*,
`candidates` : [{ `gene` : *string*, `score` : *real* }] }] }].

Note that the clinical programs of this use case mimic scenarios that arise from web-based data integration in a clinical setting. Each program adds on a level of complexity - exploring the effects of grouping, joining, and aggregating nested data in a setting that is more exploratory than a research-based analysis. The ability to manipulate these biomedical datasets within a web-based environment that supports frontend clinical exploration presents an interesting application area for the manipulation of nested collections.

Runtime performance. We execute the clinical programs with five workers, each with 20 cores and 320GB memory. We allocate 25 executors per node, 4 cores and 64GB memory per executor, 32GB memory allocated to the driver, and 1000 partitions used for shuffling data. We use the full TCGA [55] dataset (Pancancer) and the TCGA breast cancer dataset (BRCA). The pancancer dataset uses 42GB of `Occurrences` with a 10000 base flanking region, and 34GB of `CopyNumber` (34GB). The breast cancer dataset is 168 Megabytes (MB) of `Occurrences` with 10000 base flanking region and 4GB of `CopyNumber`. The runtime of a program is measured by first caching all inputs in memory.

Figure 12 shows the runtimes for each clinical program, using the standard and shredded compilation routes. We also include the results for unshredding, i.e. the cost of reconstructing the nested output when using the shredded representation. The smaller breast cancer dataset shows performance benefits of the shredded representation over flattening methods. The restructuring in `OccurGrouped` is 80x faster for shredding in comparison to standard, and still 12x as performant when the nested output type is returned. As operations are added with `OccurCNVJoin` and `OccurCNVAgg`, the shredded route exhibits up to 9x performance benefits of Standard. The results of this experiment show that the shredded compilation method can bring major advantages, even for small-scale datasets.

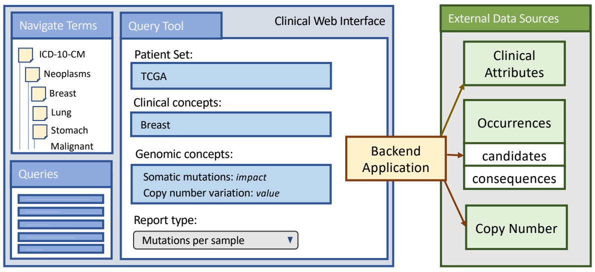

Figure 11. Mock-up of a clinical interface (i2b2) that enables integrative querying of clinical and genomic attributes.

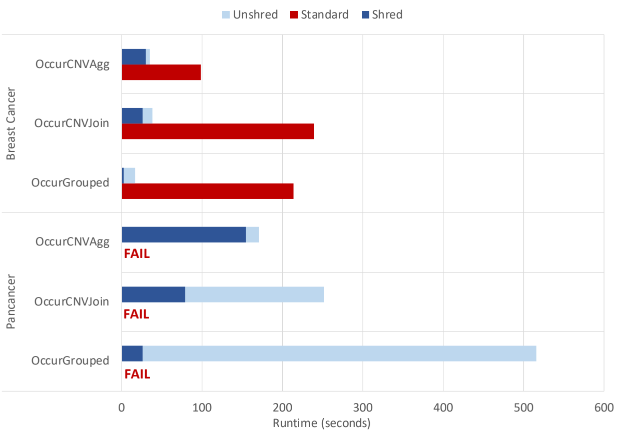

Figure 12. Results for the clinical exploration programs. The standard compilation route fails for all runs with the Pancancer dataset.

For the larger sample set, the results show that flattening methods are unable to scale, overloading the available memory on the system during each program execution. The results of Shred highlight benefits to the shredded representation. The pancancer `OccurGrouped` is very cheap, but becomes more expensive when the nested output is reconstructed (Unshred); this suggests that the succinct representation used in shredding is essential for scaling. On the other hand, more work is done during the execution of the shredded `OccurCNVAgg` program, which reduces the cost of unshredding to 3x that of `OccurGrouped`. *These results highlight how aggregation in shredded programs can bring further benefits to an analysis even when the output is returned in nested form.*

Sharing in the shredded representation

All the use cases in this section use an `Occurrences` input that is based on the occurrences endpoint of the ICGC data data portal [23], which returns JSON-formatted data following the structure of (3). In this representation, annotations will be repeated within the nested `candidates` collection for mutations that are shared across samples. We can exploit this sharing to create an even more succinct shredded representation of the `Occurrences` data source.

With all somatic mutations are in the `Mutations` data source and all unique annotations in the `Annotations` data source, we can write the following

program to construct the data returned from the occurrences endpoint:

```
BuildOccur ⇐
for m in Mutations union
 for v in Annotations union
  if m.contig == v.contig && m.start == v.start
    && m.alternate == v.alternate then
   [{sid := m.sid, mutationId := v.mutationId, ...,
    candidates := v.candidates}]}]
```

The output type of the `BuildOccur` program matches that of `Occurrences`, presented at (3). The ellipses in the `BuildOccur` program include the additional top-level fields from `Mutations` and `Annotations`. This program describes the construction of the `Occurrences` data source.

Sharing experiment. To explore the benefits of sharing, we execute the above `BuildOccur`program using the standard and shredded compilation routes. We use one somatic mutation (MAF) file from the breast cancer dataset (`Mutations`) containing 120988 tuples, and the associated unique set of 58121 VEP annotations (`Annotations`).

The association in the `BuildOccur` program translates to a join in the compiled Spark application. When the somatic mutations are joined with annotations in the standard route, the result contains 5170132 tuples nested within the `candidates` collections of the whole output. For the shredded route, the somatic mutations are joined with the top-level source `Annotations_top`, which has replaced the `candidates` values with labels. The first-level output `BuildOccur_cands` is the same as the input `Annotations_candidates`, which has 3777092 tuples. The shredded representation reduces the total size of the transcripts by over one million tuples.

The results of this experiment are based off a small dataset. Since many of the samples will share mutations specific to cancer, the benefits of sharing will increase for datasets that include more samples. To further explore the benefits of sharing by the shredded compilation route, future experiments should perform the use cases of this section with the output of `BuildOccur` in place of the `Occurrences` data source.

Conclusions

The TraNCE framework provides a foundation for exploring how query compilation and shredding optimizations can support scalable processing of nested collections. We present several use cases that highlight how the framework can support multi-modal biomedical analyses in research and clinical settings. The results show that the platform has promise in automating the challenges that arise for large-scale distributed processing of nested collections; showing scalable performance for increasing number of genomic variants and performance when flattening methods are unable to perform at all. Further, we exhibit how data integration tasks can feed into machine learning tasks and analytics pipelines. The framework is experimental and its development is ongoing, but our work shows that the techniques applied can provide a basis for many biomedical data integration tasks.

Future work should examine the interface between learning analyses and data integration tasks. For example, a user should be able to describe inference-based tasks within their programs with an extended language that supports iteration and user-defined functions.

The clinical exploration programs present an interesting perspective for the design of biomedical data integration infrastructure. Web-based data types are often nested, and our results show that manipulation of these structures using the standard flattening methods scales poorly. All the use cases have highlighted major advantages for the shredded representation, supporting nested data without compromising the ability to scale. The ability of biomedical systems and analysis applications to work on a succinct representation could present interesting opportunities for optimization, but requires adjustments in backend applications. For example, the clinical exploratory queries could display somatic mutation and copy number data in integrated format to the user, while persisting the shredded representations in the backend. A subsequent request for clinical report generation could use cached inputs that perform localized aggregate operations and return likelihood measurements or risk scores. Future work should consider situations where iterative exploration and aggregation occurs on the data, which is applicable to both research and clinical applications. We have on-going work in developing frontend interfaces focused on translations from other languages and web-based interfaces suitable for users less comfortable with writing data science applications.

Outside of clinical settings, consortium and data biobanks could consider using shredded representations in the backend. Datasets often occur as dump files, which have already gone through a pre-processing phase that employs flattening. Adapting the data representation could support the development of optimized data analysis pipelines. Overall, the TraNCE framework presents an interesting angle for systems development of research and clinical biomedical applications at scale.

Availability of source code and requirements

- Project name: TraNCE (TRAnsforming Nested Collections Efficiently)
- Project home page: `github.com/jacmarjorie/trance`
- Operating system(s): Platform independent
- Programming language: Scala 2.12
- Other requirements: Spark
- License: MIT
- RRID: SCR_021252

Data availability

TraNCE is registered with bio.tools at `bio.tools/trance` and SciCrunch RRID:SCR_021252. The data set supporting the results of this article, primarily raw runtimes of performance results, are available in the figshare repository at `doi.org/10.6084/m9.figshare.13363502`. All supporting data and materials are available in the GigaScience GigaDB database [44].

Declarations

List of abbreviations

API: Application Programming Interface; BRCA: Breast Cancer; CNV: Copy Number Variation; DLBC: Lymphoid Neoplasm Diffuse Large B-cell Lymphoma; FPKM: Fragments Per Kilobase of transcript per Million mapped read; EHR: Electronic Health Record; GB: Gigabyte; GDC: Genomic Data Commons; GMB: Gene Mutational Burden; GTF: General Transfer Format; i2b2: Informatics for Integrating Biology and Bedside; ICGC: International Genome Consortium; ID: Identifier; JSON: JavaScript Object Notation; MAF: Mutation Annotation Format; MB: Megabyte; MSigDB: The Molecular Signatures Database; NRC: Nested Relational Calculus; PMB: Pathway Mutational Burden; RDD: Resilient Distributed Dataset; SO: Sequence Ontology; SQL: Structured Query Language; TCGA: The Cancer Genome Atlas; TMB: Tumor Mutational Burden; TraNCE: Transforming Nested Collections Efficiently; VCF: Variant Call Format; VEP: Variant Effect Predictor;

Consent for publication

Not applicable.

Competing Interests

The author(s) declare that they have no competing interests.

Funding

The work was funded by EPSRC grant EP/M005852/1 and by Oxford's EPSRC IAA Technology Fund, grant EP/R511742/1.

Author's Contributions

JS, MB, and MN conceived the idea and design of the framework. JS and MN built the framework. JS and YS conceived and designed the burden based analyses; YS performed and validated the burden-based analyses. JS conceived, designed, performed, and validated the driver and clinical analyses. MB and MN supervised the project. JS wrote the original draft of the manuscript. All authors reviewed and edited the manuscript.

Acknowledgements

The authors would like to thank Omics Data Automation, Inc. for supplying hardware, compute time, and contributing to use case discussions.

References

1. Adzhubei IA, Schmidt S, Peshkin L, Ramensky VE, Gerasimova A, Bork P, et al. A method and server for predicting damaging missense mutations. Nature Methods 2010;7(4):248–249.
2. Afgan E, Baker D, Batut B, van den Beek M, Bouvier D, Čech M, et al. The Galaxy platform for accessible, reproducible and collaborative biomedical analyses: 2018 update. Nucleic Acids Research 2018;46(W1):W537–W544.
3. Armbrust M, Xin RS, Lian C, Huai Y, Liu D, Bradley JK, et al. Spark SQL: Relational Data Processing in Spark. In: SIGMOD; 2015. p. 1383–1394.
4. Auton A, Abecasis GR, Altshuler DM, Durbin RM, Abecasis GR, Bentley DR, et al. A global reference for human genetic variation. Nature 2015;526(7571):68–74.
5. Buneman P, Naqvi S, Tannen V, Wong L. Principles of programming with complex objects and collection types. Theoret Comput Sci 1995;149(1):3–48.
6. den Bussche JV. Simulation of the Nested Relational Algebra by the Flat Relational Algebra. Theor Comput Sci 2001;254(1-2):363–377.
7. Chalmers ZR, Connelly CF, Fabrizio D, Gay L, Ali SM, Ennis R, et al. Analysis of 100,000 human cancer genomes reveals the landscape of tumor mutational burden. Genome Medicine 2017;9(1):34.
8. Cheng F ZZ Zhao J. Advances in computational approaches for prioritizing driver mutations and significantly mutated genes in cancer genomes. Briefings in Bioinformatics 2016;17(4):642–656.
9. Coppola L, Cianflone A, Grimaldi AM, Incoronato M, Bevilacqua P, Messina F, et al. Biobanking in health care: evolution and future directions. Journal of Translational Medicine 2019;17(1):172.
10. Curoverse I. Introduction to Arvados A Curoverse White Paper. Curoverse, Inc.; 2014.
11. Eilbeck K, Lewis SE, Mungall CJ, Yandell M, Stein L, Durbin R, et al. The Sequence Ontology: A tool for the unification of genome annotations. Nature Methods 2005;6:R44.
12. Fancello L, Gandini S, Pelicci PG, Mazzarella L. Tumor mutational burden quantification from targeted gene panels: major advancements and challenges. Journal for ImmunoTherapy of Cancer 2019;7(1):183. https://doi.org/10.1186/s40425-019-0647-4.
13. Fegaras L, Maier D. Optimizing object queries using an effective calculus. ACM Transactions on Database Systems 2000;25(4):457–516.
14. Gabetta M, Limongelli I, Rizzo E, Riva A, Segagni D, Bellazzi R. BigQ: a NoSQL based framework to handle genomic variants in i2b2. BMC Bioinformatics 2015;16(1):415.
15. Genomic Data Commons Endpoints; 2020. https://docs.icgc.org/portal/api-endpoints/.
16. Glow; 2019. https://github.com/projectglow/glow.
17. Greenman C, Stephens P, Smith R, Dalgliesh GL, Hunter C, Bignell G, et al. Patterns of somatic mutation in human cancer genomes. Nature 2007;446(7132):153–158.
18. Hail; 2015. https://github.com/hail-is/hail.
19. He KY, Ge D, He MM. Big Data Analytics for Genomic Medicine. International journal of molecular sciences 2017 02;18(2):412.
20. Hodson R. Precision medicine. Nature 2016;537(7619):S49.
21. A Java API for high-throughput sequencing data (HTS) formats.; 2020. https://samtools.github.io/htsjdk/.
22. i2b2; 2020. i2b2.org/software/index.html.
23. International Cancer Genome Consortium; 2020. https://icgc.org/.
24. Jiao W, Atwal G, Polak P, Karlic R, Cuppen E, Al-Shahrour F, et al. A deep learning system accurately classifies primary and metastatic cancers using passenger mutation patterns. Nature Communications 2020;11(1):728. https://doi.org/10.1038/s41467-019-13825-8.
25. JK G, B TC, M J, S G, Y M, R M, et al. Application of a Neural Network Whole Transcriptome-Based Pan-Cancer Method for Diagnosis of Primary and Metastatic Cancers. JAMA Netw Open 2019 April;2(4):e192597.
26. Keras; 2020. https://keras.io/.
27. Kichaev G, Yang WY, Lindstrom S, Hormozdiari F, Eskin E, Price AL, et al. Integrating Functional Data to Prioritize Causal Variants in Statistical Fine-Mapping Studies. PLoS Genetics 2014;10(10).
28. Kingma DP, Ba J. Adam: A Method for Stochastic Optimization. arXiv 2014 Dec;https://arxiv.org/abs/1412.6980v9.
29. Liang Y, Wang H, Yang J, Li X, Dai C, Shao P, et al. A Deep Learning Framework to Predict Tumor Tissue-of-Origin Based on Copy Number Alteration. Frontiers in Bioengineering and Biotechnology 2020;8:701. frontiersin.org/article/10.3389/fbioe.2020.00701.
30. Masseroli M, Pinoli P, Venco F, Kaitoua A, Jalili V, Palluzzi F, et al. GenoMetric Query Language: A Novel Approach to Large-scale Genomic Data Management. Bioinformatics 2015;31(12):1881–1888.
31. Massie M, Nothaft F, Hartl C, Kozanitis C, Schumacher A, Joseph AD, et al. ADAM: Genomics Formats and Processing Patterns for Cloud Scale Computing. UCB/EECS; 2013.
32. McLaren W, Gil L, Hunt SE, Riat HS, Ritchie GRS, Thormann A, et al. The Ensembl Variant Effect Predictor. Genome Biology 2016;17(1):122.
33. Mootha VK, Lindgren CM, Eriksson KF, Subramanian A, Sihag S, Lehar J, et al. PGC-1alpha-responsive genes involved in oxidative phosphorylation are coordinately downregulated in human diabetes. Nature Genetics 2003;34(3):267–273.
34. Murphy SN, Avillach P, Bellazzi R, Phillips L, Gabetta M, Eran A, et al. Combining clinical and genomics queries using i2b2 – Three methods. PLOS ONE 2017 04;12(4):1–16.
35. Nothaft FA, Massie M, Timothy D, Zhang Z, Laserson U, Yeksigian C, et al. Rethinking Data-Intensive Science Using Scalable Analytics Systems. In: SIGMOD; 2015. .
36. Oinn T, Addis M, Ferris J, Marvin D, Senger M, Greenwood M, et al. Taverna: A tool for the composition and enactment of bioinformatics workflows. Bioinformatics 2004;20(17):3045–3054.
37. Pezoa F, Reutter JL, Suarez F, Ugarte M, Vrgoč D. Foundations of JSON schema. In: WWW; 2016. .
38. PySpark; 2020. Online documentation.
39. scikit-learn; 2020. https://scikit-learn.org/stable/.
40. Smemo S, Tena JJ, Nóbrega MA. Obesity-associated variants within FTO form long-range functional connections with IRX3. Nature 2014;507(7492):371–375.
41. Smith J, Benedikt M, Nikolic M, Shaikhha A, Scalable Querying of Nested Data; 2020. arxiv.org/abs/2011.06381.
42. Smith J, Benedikt M, Nikolic M, Shaikhha A, Scalable Querying of Nested Data; 2020. github.com/jacmarjorie/trance.
43. Smith J, Benedikt M, Nikolic M, Shaikhha A. Scalable Querying of Nested Data. In: VLDB; 2021. .
44. Smith J, Shi Y, Benedikt M, Nikolic M. Supporting data for "Scalable Analysis of Multi-Modal Biomedical Data". GigaScience Database 2021;http://dx.doi.org/10.5524/100914.
45. Smith JM, Lathara M, Wright H, Hill B, Ganapati N, Srinivasa G, et al. Advancing clinical cohort selection with genomics analysis on a distributed platform. PLOS ONE 2020 04;15(4):1–20.
46. Srivastava N, Hinton G, Krizhevsky A, Sutskever I, Salakhutdinov R. Dropout: a simple way to prevent neural networks from overfitting. Journal of Machine Learning Research 2014;15(1):1929–1958.
47. Subramanian A, Tamayo P, Mootha VK, Mukherjee S, Ebert BL, Gillette MA, et al. Gene set enrichment analysis: a knowledge-based approach for interpreting genome-wide expression profiles. Proc Natl Acad Sci USA

2005;102(43):15545–15550.

48. Sudlow C, Gallacher J, Allen N, Beral V, Burton P, Danesh J, et al. UK Biobank: An Open Access Resource for Identifying the Causes of a Wide Range of Complex Diseases of Middle and Old Age. PLoS Medicine 2015;12(3):1–10.
49. Sudmant PH, Rausch T, Gardner EJ, Handsaker RE, Abyzov A, Huddleston J, et al. An integrated map of structural variation in 2,504 human genomes. Nature 2015;526(7571):75–81.
50. Szklarczyk D, Gable AL, Lyon D, Junge A, Wyder S, Huerta-Cepas J, et al. STRING v11: protein-protein association networks with increased coverage, supporting functional discovery in genome-wide experimental datasets. Nucleic Acids Res 2019;47(D1):D607–D613.
51. pandas development team T, pandas-dev/pandas: Pandas. Zenodo; 2020. https://doi.org/10.5281/zenodo.3509134.
52. Vaser R, Adusumalli S, Leng SN, Sikic M, Ng P. SIFT missense predictions for genomes. Nature Protocols 2009;11(1):1073–1081.
53. Voss K, Gentry J, Auwera GVD. Full-stack genomics pipelining with GATK4+ WDL+ Cromwell [version 1; not peer reviewed]. F1000Research 2017;p. 4.
54. Wang Q, Xu M, Sun Y, Chen J, Chen C, Qian C, et al. Gene Expression Profiling for Diagnosis of Triple-Negative Breast Cancer: A Multicenter, Retrospective Cohort Study. Front Oncol 2019 May;9:354.
55. Weinstein JN, Collisson EA, Mills GB, Shaw KM, Brad A, Ellrott K, et al. The Cancer Genome Atlas Pan-Cancer Analysis Project. Nature Genetics 2013;45(10):1113–1120.
56. Wong L. Querying Nested Collections. PhD dissertation, University of Pennsylvania; 1994.
57. Xu B, Wang N, Chen T, Li M. Empirical Evaluation of Rectified Activations in Convolutional Network. arXiv 2015 May;https://arxiv.org/abs/1505.00853v2.
58. Zaharia M, Chowdhury M, Das T, Dave A, Ma J, McCauly M, et al. Resilient Distributed Datasets: A Fault-Tolerant Abstraction for In-Memory Cluster Computing. In: 9th USENIX Symposium on Networked Systems Design and Implementation (NSDI 12); 2016. .
59. Zaharia M, Chowdhury M, Franklin MJ, Shenker S, Stoica I. Spark: Cluster Computing with Working Sets. In: 2nd USENIX Workshop on Hot Topics in Cloud Computing, HotCloud'10; 2010. .
60. Zhang W, Wang SL. A Novel Method for Identifying the Potential Cancer Driver Genes Based on Molecular Data Integration. Biochemical Genetics 2020;58(1):16–39.
61. Zhao X, Guan S, Man KL. An Output Grouping Based Approach to Multiclass Classification Using Support Vector Machines. In: Park JJJH, Jin H, Jeong YS, Khan MK, editors. Advanced Multimedia and Ubiquitous Engineering Singapore: Springer Singapore; 2016. p. 389–395.
62. Zheng Y, Ding Y, Wang Q, Sun Y, Teng X, Gao Q, et al. 90-gene signature assay for tissue origin diagnosis of brain metastases. Journal of translational medicine 2019;17(1):331.

Figure 1 included in PDF

Click here to access/download;Figure;figure1.pdf

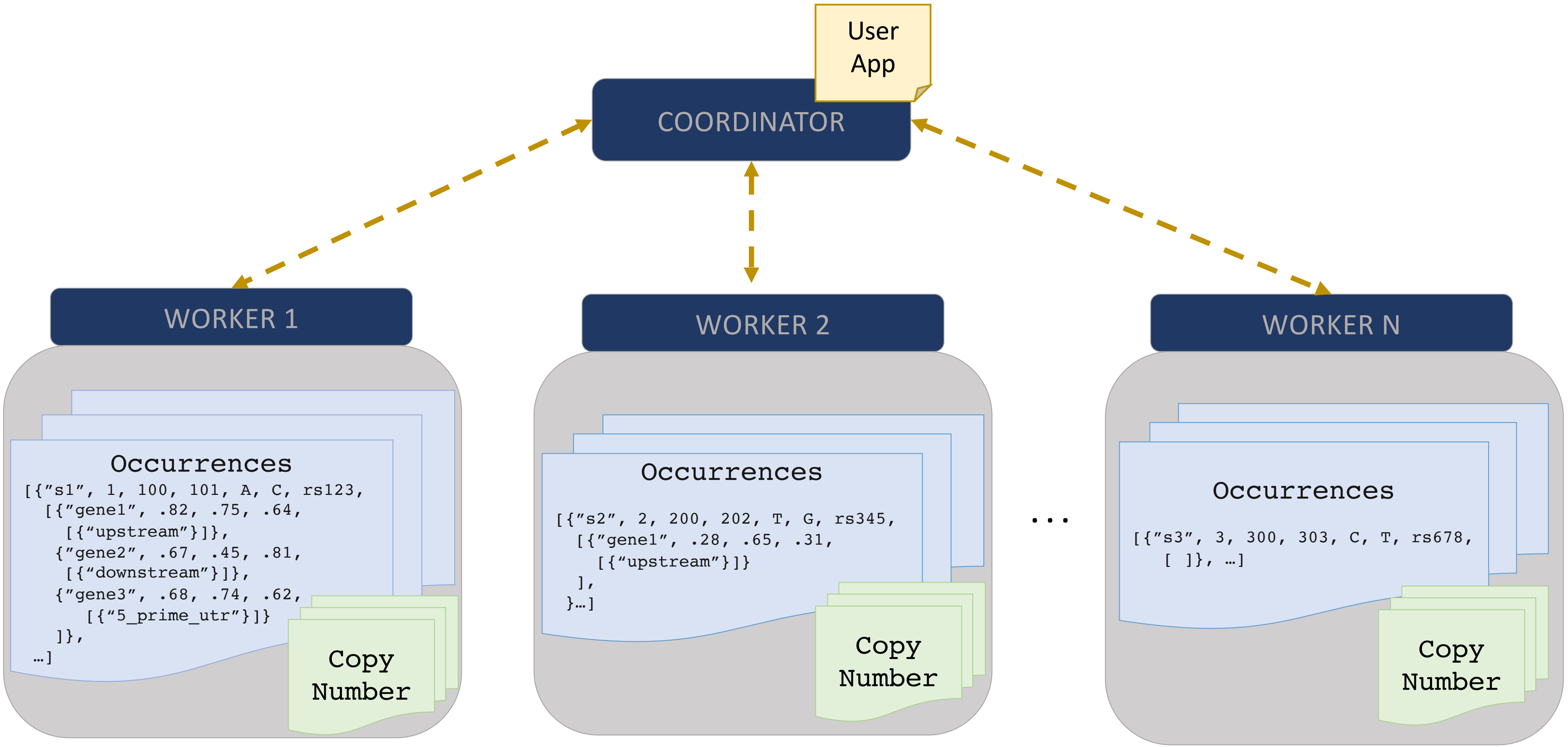

Figure 4 included in PDF

Click here to access/download;Figure;figure4.pdf

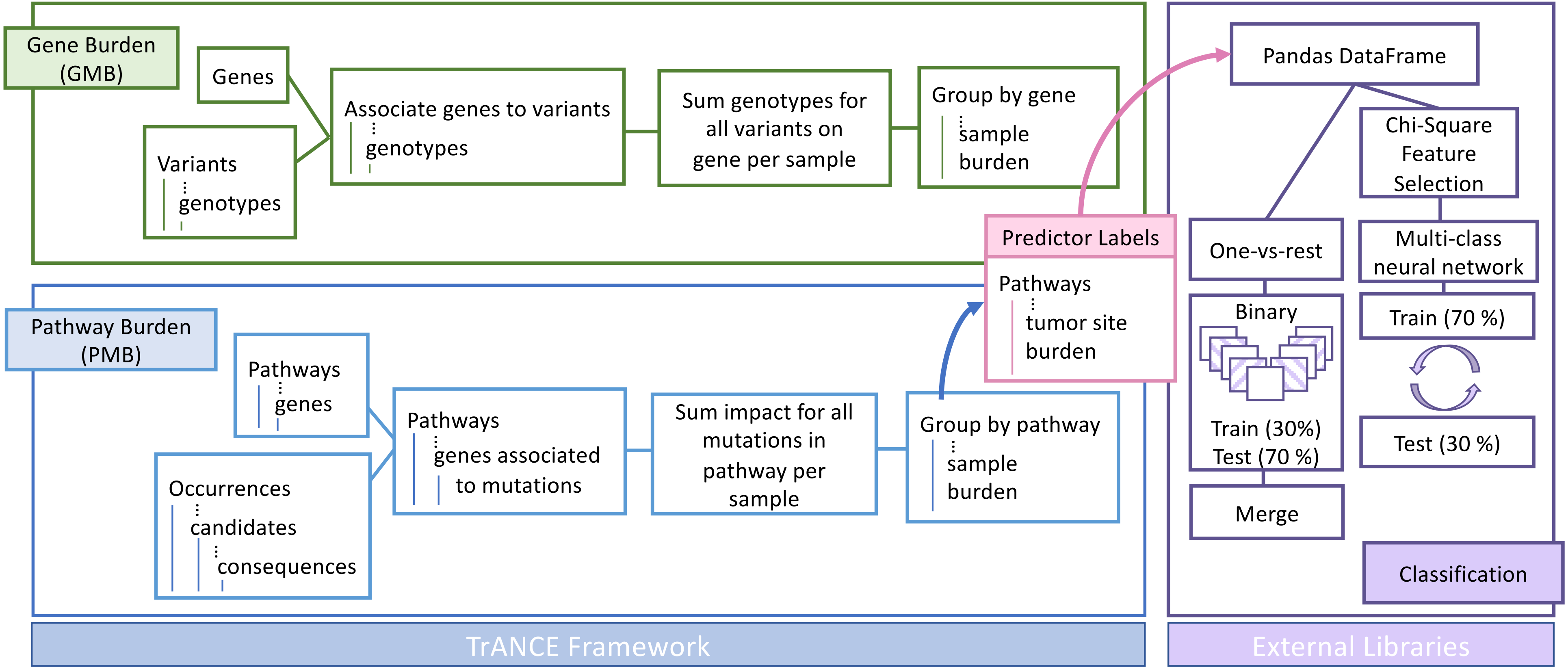

Figure 5 included in PDF

Click here to access/download;Figure;figure5.pdf

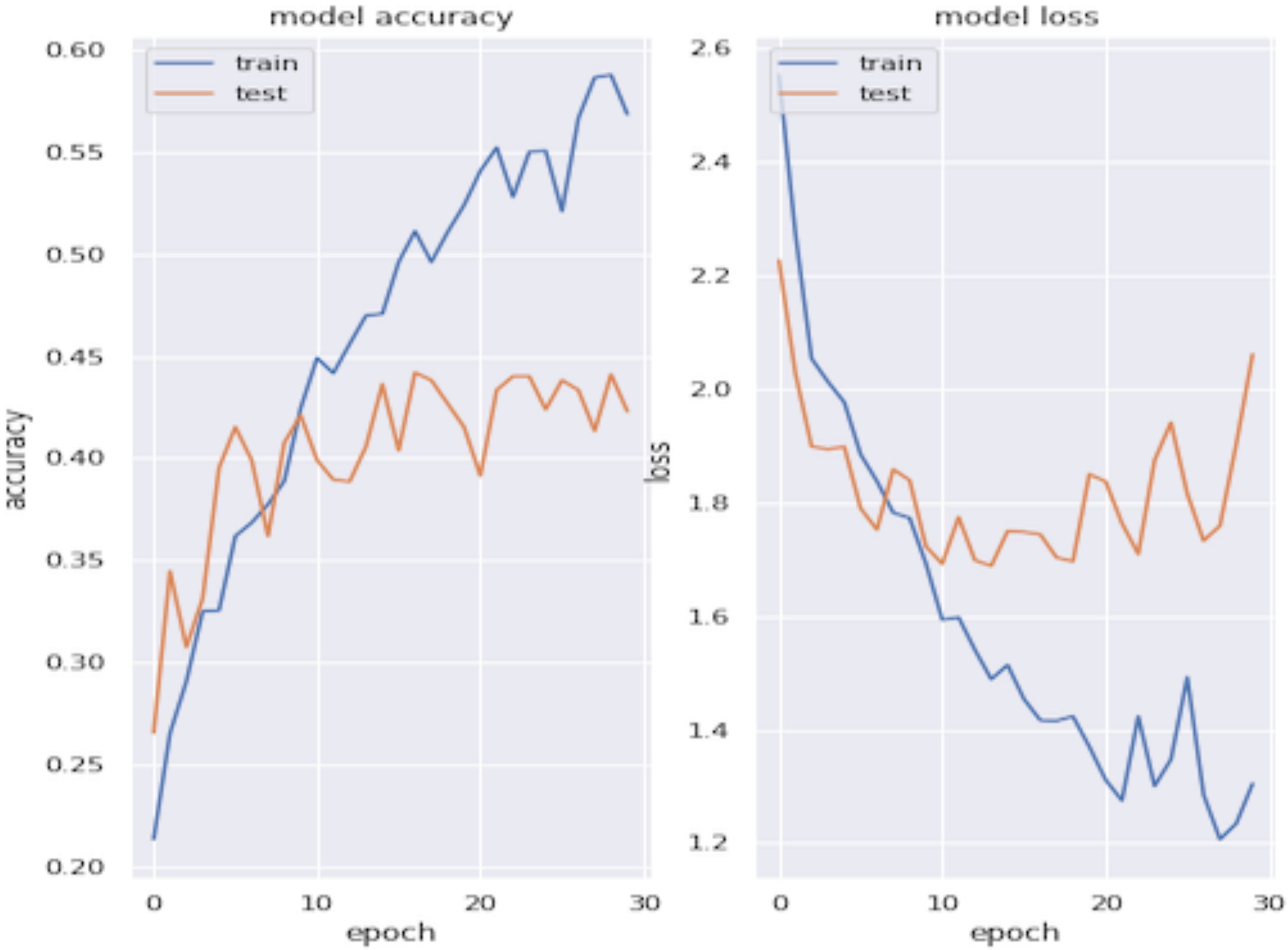

Figure 6 included in PDF
Click here to access/download;Figure;figure6.

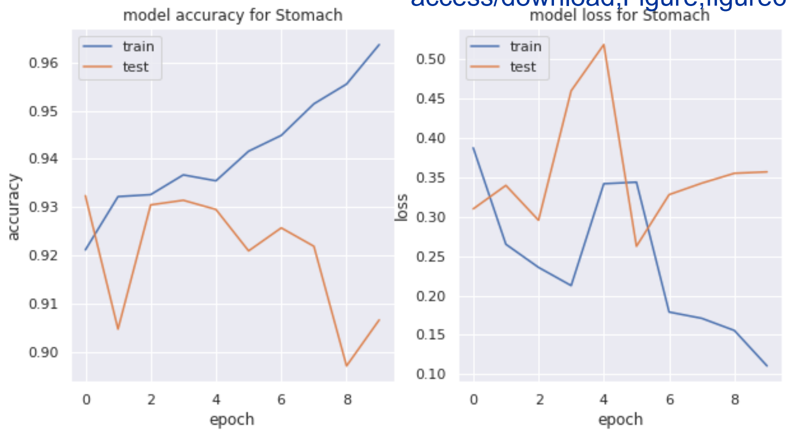

(a) Stomach

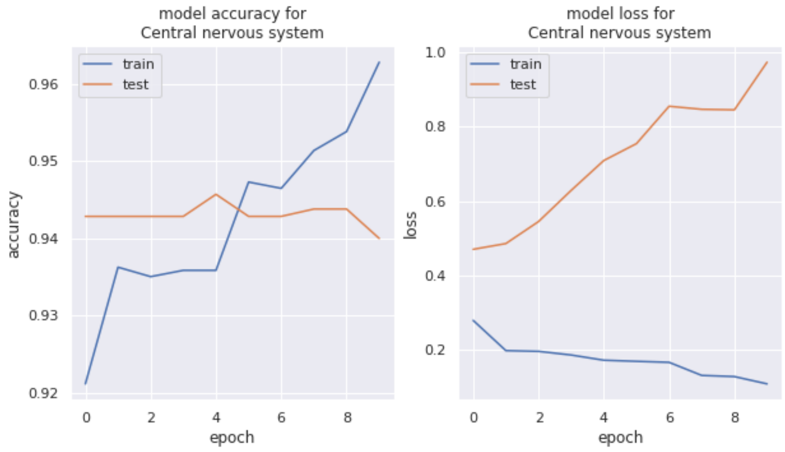

(b) Central nervous system

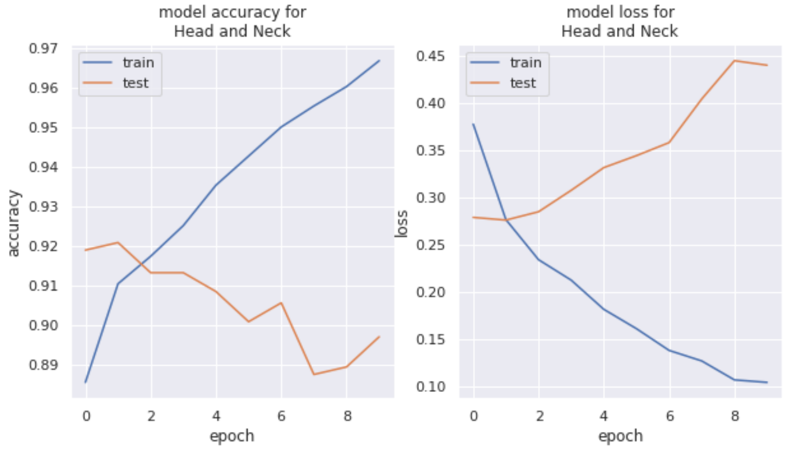

(c) Head and neck

Figure 7 included in PDF
Click here to access/download;Figure;figure7.pdf

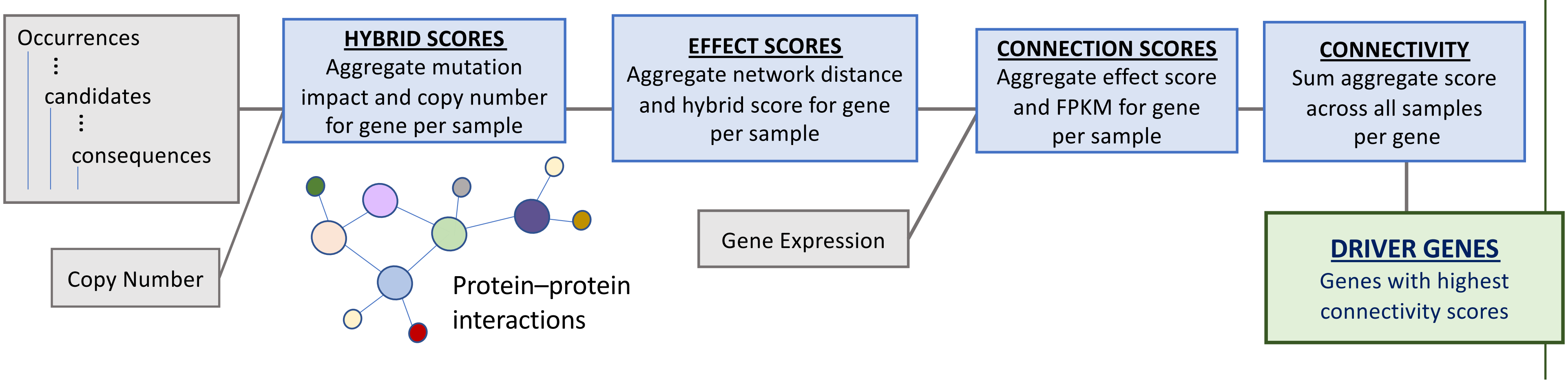

Figure 8 included in PDF Click here to access/download;Figure;figure8.pdf

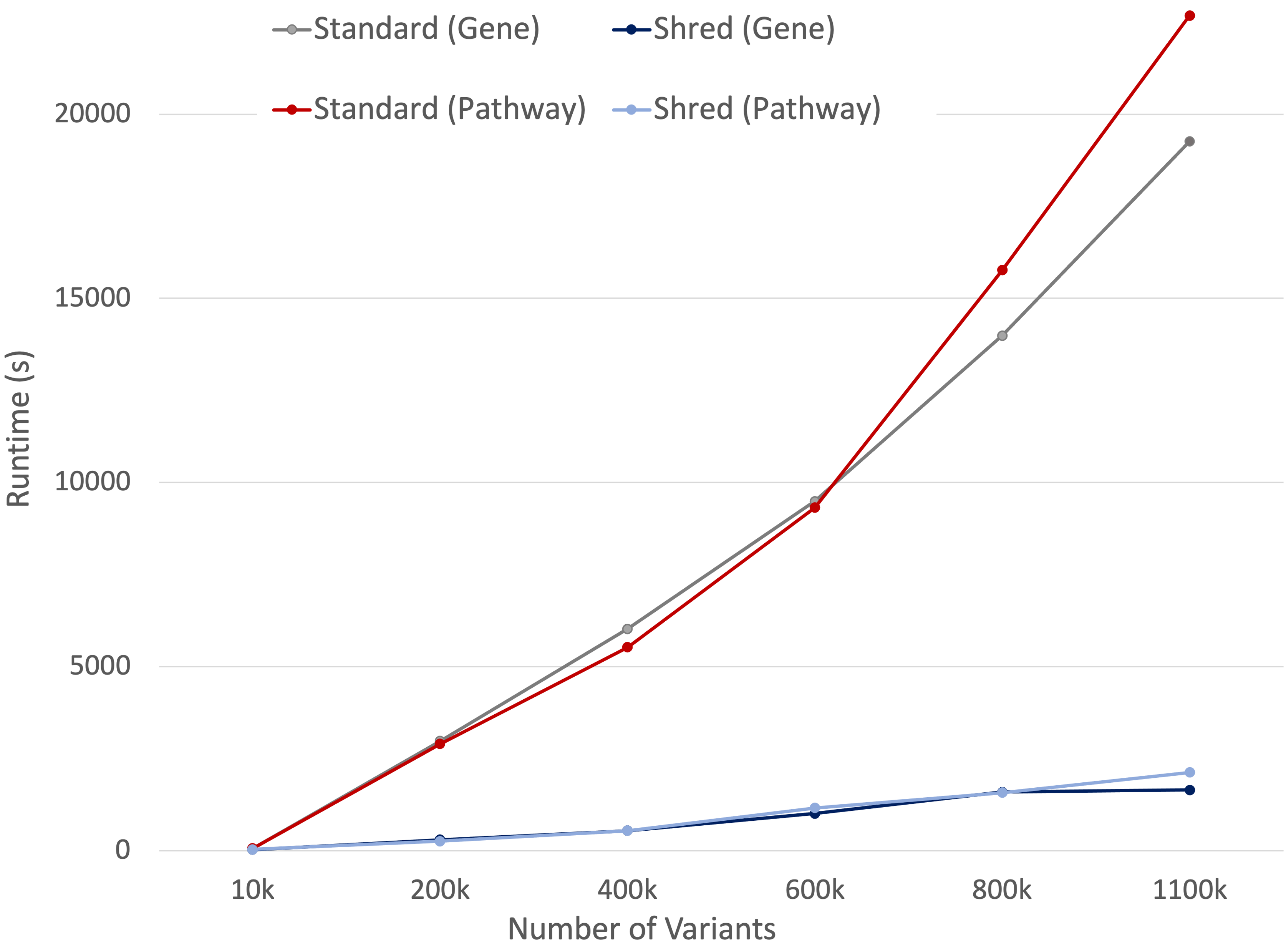

Figure 9 included in PDF

Click here to access/download;Figure;figure9.pdf

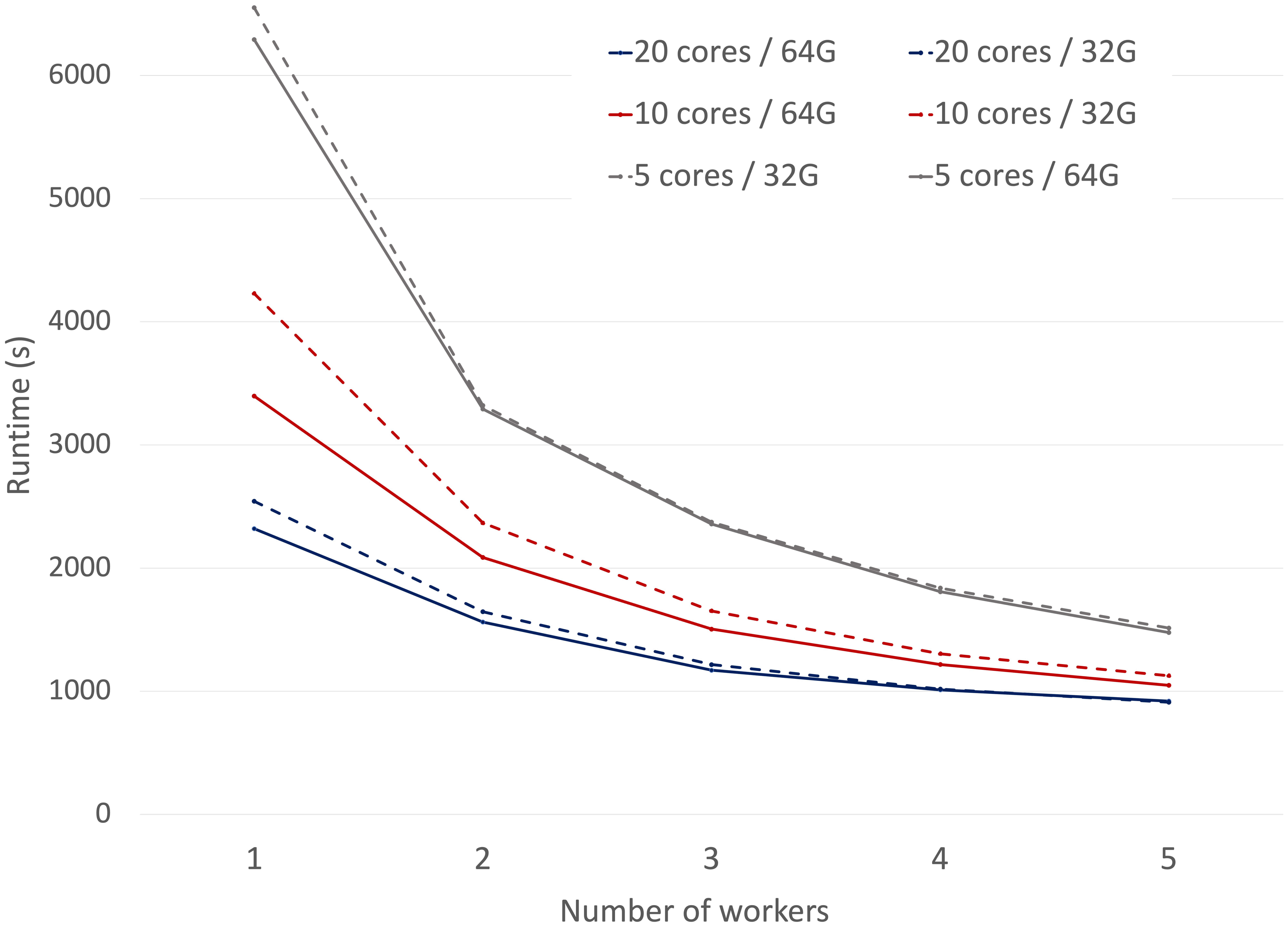

Figure 10 included in PDF Click here to access/download;Figure;figure10.pdf

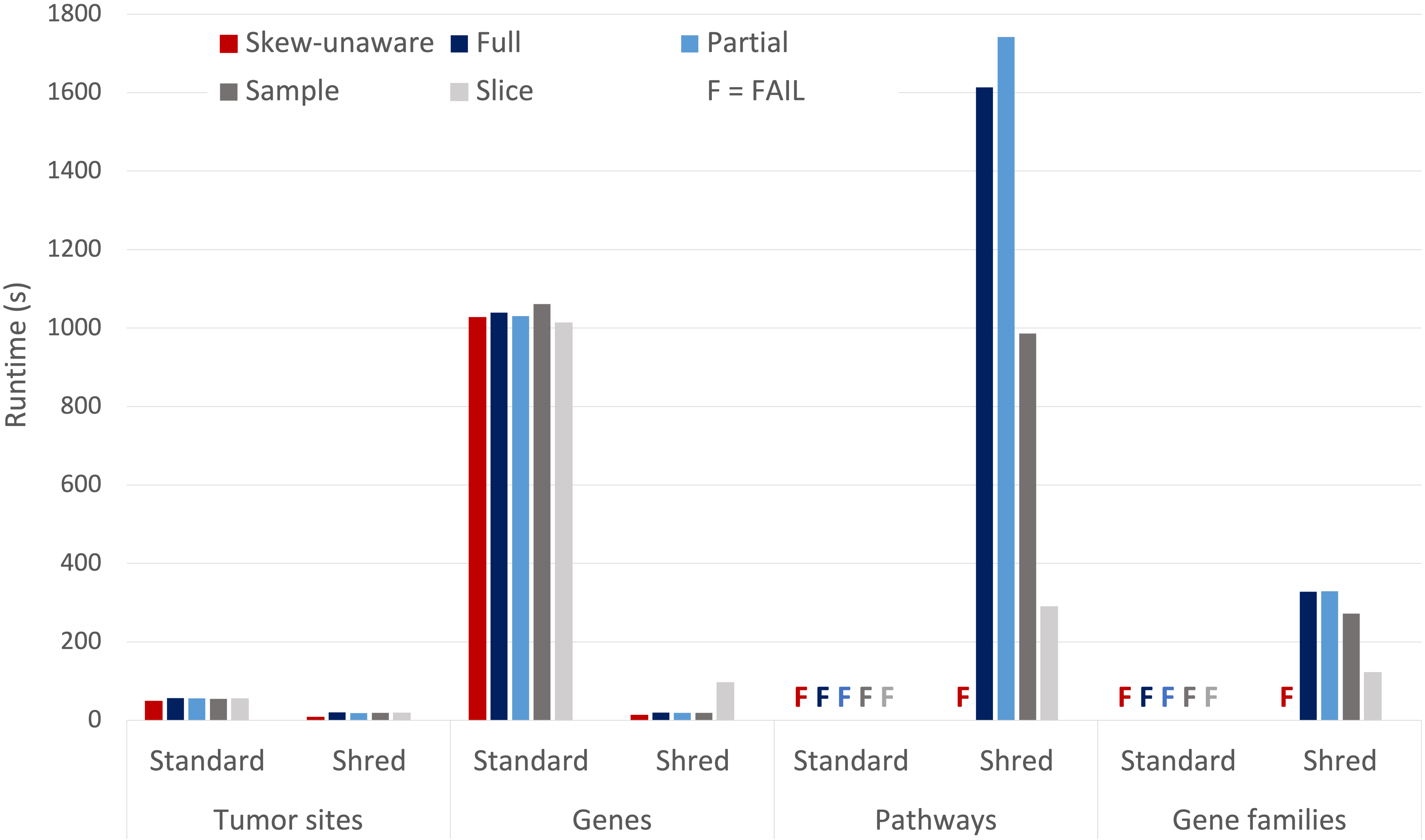

Figure 11 included in PDF Click here to access/download;Figure;figure11.pdf

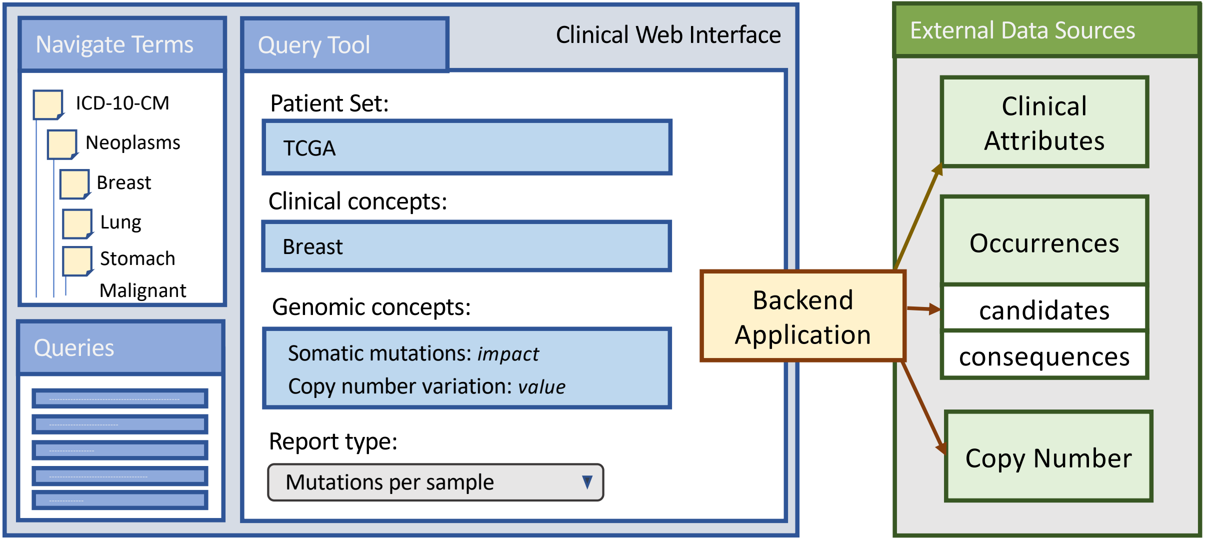

Figure 12 included in PDF

Click here to access/download;Figure;figure12.pdf

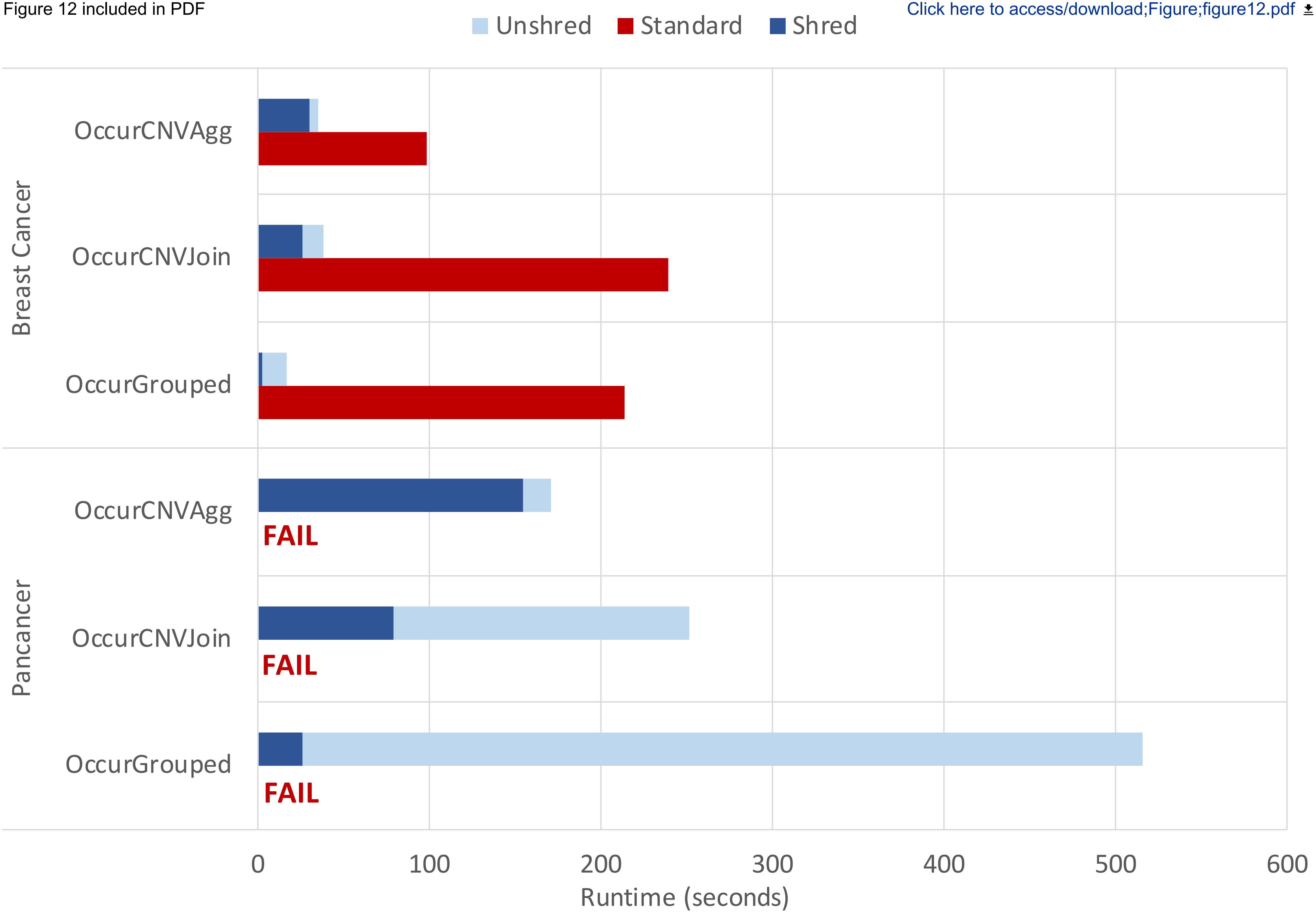

Click here to access/download
Supplementary Material
supplementary.pdf

Click here to access/download
Supplementary Material
response.pdf
