# Supplementary material for: Scalable analysis of multi-modal biomedical data
Source: Gigascience. 2021 Sep 11;10(9):giab058. doi: 10.1093/gigascience/giab058 (PMC8434767; doi:10.1093/gigascience/giab058)
Supplement: giab058_Supplemental_Files [file giab058_supplemental_files.zip › supplementary.pdf]

1 Input data sources

Here, we describe and provide the datatypes for all the input sources used in the programs of the main body of the paper.

1.1 Copy Number

The copy number variation (CNV) data source returns by-gene copy number information for each `sid`; this is the number of copies of a particular gene measured in a sample. The type of copy number information is:

$$[\ \{\ \texttt{sid} : \mathit{string}, \texttt{gene} : \mathit{string}, \texttt{cnum} : \mathit{int}\ \}\ ]. \tag{1}$$

1.2 Occurrences

The `Occurrences` data source returns somatic mutations and associated annotation information for each sample. An occurrence represents a single, annotated mutation belonging to a single sample. The type of `Occurrences` is:

$$\begin{aligned}&[\ \{\ \texttt{sid} : \mathit{string}, \texttt{contig} : \mathit{string}, \texttt{start} : \mathit{int}, \texttt{end} : \mathit{int},\\ &\quad \texttt{reference} : \mathit{string}, \texttt{alternate} : \mathit{string}, \texttt{mutationId} : \mathit{string},\\ &\quad\quad \texttt{candidates} :\ [\ \{\ \texttt{gene} : \mathit{string}, \texttt{impact} : \mathit{string},\\ &\quad\quad\quad \texttt{sift} : \mathit{real}, \texttt{poly} : \mathit{real},\\ &\quad\quad\quad \texttt{consequences} :\ [\ \{\ \texttt{conseq} : \mathit{string}\ \}\ ]\ \}\ ]\ \}\ ].\end{aligned} \tag{2}$$

The attribute `candidates` identifies a collection of objects that contain attributes corresponding to the predicted effects a mutation has on a gene; i.e. *variant annotations* sourced from the Variant Effect Predictor (VEP) [?]. The `impact` attribute is a value from 0 to 1 denoting the estimated consequence a mutation has to a gene based on sequence conservation. The `sift` and `poly` attributes provide additional impact scores determined from the Sift [?] and PolyPhen [?] prediction software. These scores estimate the influence a mutation has on functional changes to proteins based on amino acid substitution. The `consequences` for each candidate gene contain categorical assignments of mutation impact based on sequence ontology (SO) terms [?].

VEP provides a distance flag that specifies the upstream and downstream range used to identify gene-based annotations (i.e. the flanking region). This distance flag specifies the size of `candidates`, since more genes are assigned as candidates with a larger flanking regions. A larger value can be used to determine long-range functional connections.

1.3 Variants

The `Variants` data source is based the VariantContext [?] object, used to represent variants from a Variant Call Format (VCF) file. This data structure represents

one line, i.e. one variant, from a VCF file. Variants are identified by chromosome, position, reference and alternate alleles, and associated genotype information for every sample. We use an integer-based categorical assignment to genotype calls to support analyses; 0 is homozygous reference with no mutated alleles, 1 is heterozygous with 1 mutated allele, and 2 is homozygous alternate with 2 mutated alleles. The type of `Variants` is:

$$[\ \{\ \texttt{contig} : \textit{string}, \texttt{start} : \textit{int}, \texttt{reference} : \textit{string}, \texttt{alternate} : \textit{string}, \texttt{genotypes} :\ [\ \{\ \texttt{sid} : \textit{string}, \texttt{call} : \textit{int}\ \}\ ]\ \}\ ].$$

1.4 Somatic mutations

Somatic mutations are stored in the GDC in Mutation Annotation Format (MAF), which is a flat datadump that includes a line for every mutation across all samples. The type of `Mutations` is:

$$[\ \{\ \texttt{sid} : \textit{string}, \texttt{contig} : \textit{string}, \texttt{start} : \textit{int}, \texttt{end} : \textit{int}, \texttt{reference} : \textit{string}, \texttt{alternate} : \textit{string}, \texttt{mutationId} : \textit{string}\ \}\ ]$$

1.5 Variant Annotations

The variant annotations come from the VEP software, which takes as input mutation information either in VCF or MAF format and returns top-level mutation information augmented with two additional levels of gene and mutational impact information. The overall structure is similar to the `Occurrences` data source (2), except VEP returns a unique set of variant annotations that are not associated to a specific sample. The type of the `Annotations` data source is:

$$[\ \{\ \texttt{contig} : \textit{string}, \texttt{start} : \textit{int}, \texttt{end} : \textit{int}, \texttt{reference} : \textit{string}, \texttt{alternate} : \textit{string}, \texttt{mutationId} : \textit{string}, \texttt{candidates} :\ [\ \{\ \texttt{gene} : \textit{string}, \texttt{impact} : \textit{string}, \texttt{sift} : \textit{real}, \texttt{poly} : \textit{real}, \texttt{consequences} :\ [\ \{\ \texttt{conseq} : \textit{string}\ \}\ ]\ \}\ ]\ \}\ ].$$

1.6 Protein-protein interaction network

This `Network` input is derived from the STRING [?] database, which provides a likelihood score of two proteins interacting in a system. The network is represented with a top-level node object and a nested bag of edges. Each edge object contains an edge protein and a set of node-edge relationship measurements. The type of `Network` is:

$$[\ \{\ \texttt{nodeProtein} : \textit{string}, \texttt{edges} :\ [\ \{\ \texttt{edgeProtein} : \textit{string}, \texttt{distance} : \textit{int}\ \}\ ]\ \}\ ].$$

1.7 Gene expression

Gene expression measurements are derived by comparing transcript counts in an `aliquot` to a reference count. The expression measurement is a normalized count, Fragments Per Kilobase of transcript per Million mapped read (FPKM). The type of `GeneExpression` is:

$$[\ \{\ \texttt{aliquot} : \textit{string}, \texttt{gene} : \textit{string}, \texttt{fpkm} : \textit{real}\ \}\ ].$$

1.8 Pathway

Pathways are represented as a set of genes. Pathway information is downloaded as a list of curated gene sets from The Molecular Signatures Database (MSigDB) [?, ?]. The type of `Pathway` is:

$$[\ \{\ \texttt{pathway} : \textit{string}, \texttt{genes} :\ [\ \{\ \texttt{gene} : \textit{string}\ \}\ ]\ \}\ ].$$

1.9 Sample Metadata

The `Samples` input maps samples to their aliquots; for the sake of these use cases `sid` maps to a patient and `aliquot` associates each biological sample taken from the patient. Note that this is an extend version of `Samples` introduced at (??). The type of `Samples` is:

$$[\ \{\ \texttt{sid} : \textit{string}, \texttt{aliquot} : \textit{string}, \texttt{tumorsite} : \textit{string}\ \}\ ].$$

1.10 Sequence Ontology

The `SOImpact` input is a table derived from the sequence ontology [?] that maps a qualitative consequence to a quantitative consequence score (`conseq`). This is a continuous measurement from 0 to 1, with larger values representing more detrimental consequences. The type of `SOImpact` is:

$$[\ \{\ \texttt{conseq} : \textit{string}, \texttt{value} : \textit{real}\ \}\ ].$$

1.11 Biomart Gene Map

The `Biomart` gene map input is exported from [?]. It is a map from gene identifiers to protein identifiers. This map is required to associate genes from `Occurrences` and `CopyNumber` to proteins that make up `Network`. The type `Biomart` is:

$$[\ \{\ \texttt{gene} : \textit{string}, \texttt{protein} : \textit{string}\ \}\ ].$$

1.12 Positional Gene Map

Gene mapping files provide the positional location of a gene on a genome, which is a combination of chromosome, start, and end position provided from a General Transfer Format (GTF) file. Each line of the GTF file maps a gene with its positional information [?]. The GTF file can be represented as a flat collection, `Genes`, with type:

$$[\ \{\ \texttt{gene} : \textit{string}, \texttt{description} : \textit{string}, \texttt{contig} : \textit{string}, \\ \texttt{gid} : \textit{string}, \texttt{start} : \textit{int}, \texttt{end} : \textit{int}, \texttt{name} : \textit{string}\ \}\ ].$$

The above data sources are referenced throughout the use cases in the subsections that follow.

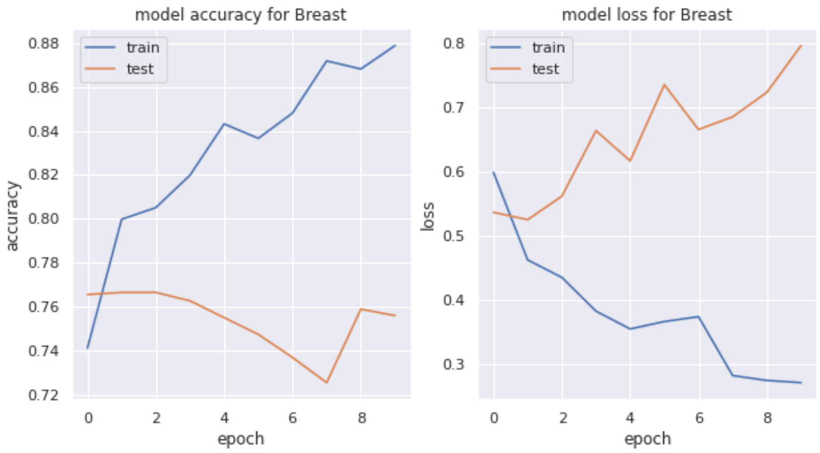

Figure 1: The accuracy and loss of the binary neural network for breast.

2 Binary models

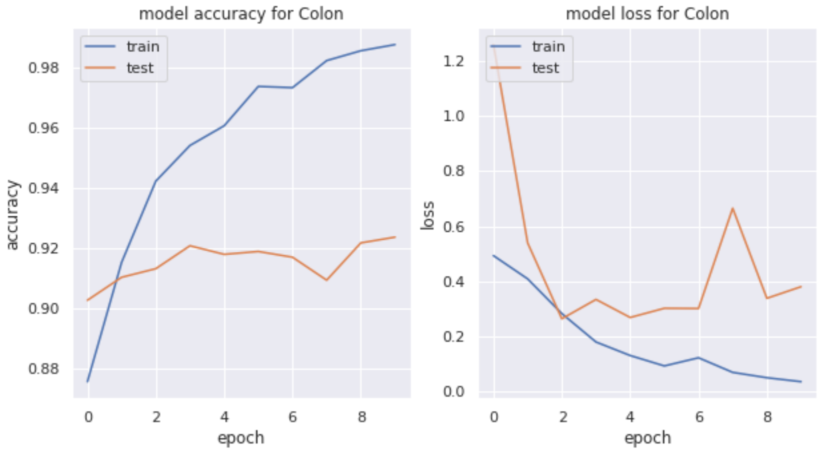

Figure 2: The accuracy and loss of the binary neural network for colon.

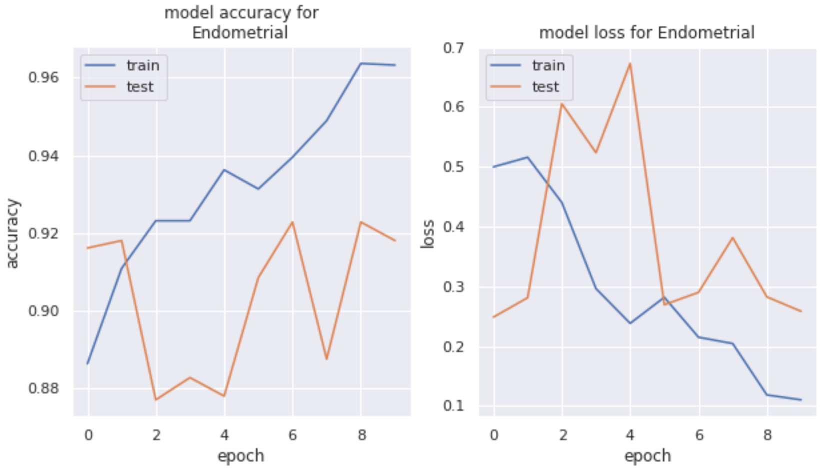

Figure 3: The accuracy and loss of the binary neural network for endometrial.

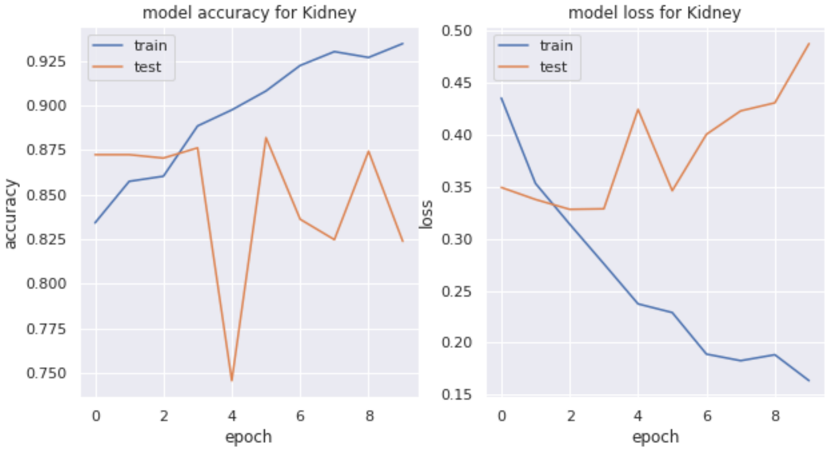

Figure 4: The accuracy and loss of the binary neural network for kidney.

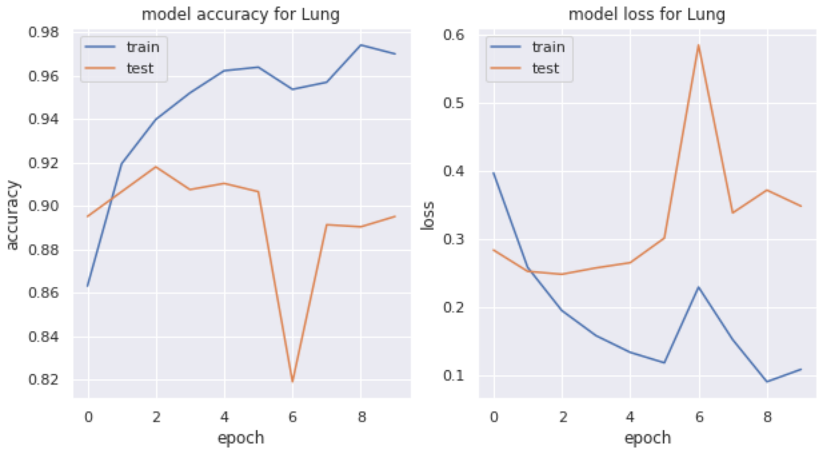

Figure 5: The accuracy and loss of the binary neural network for lung.

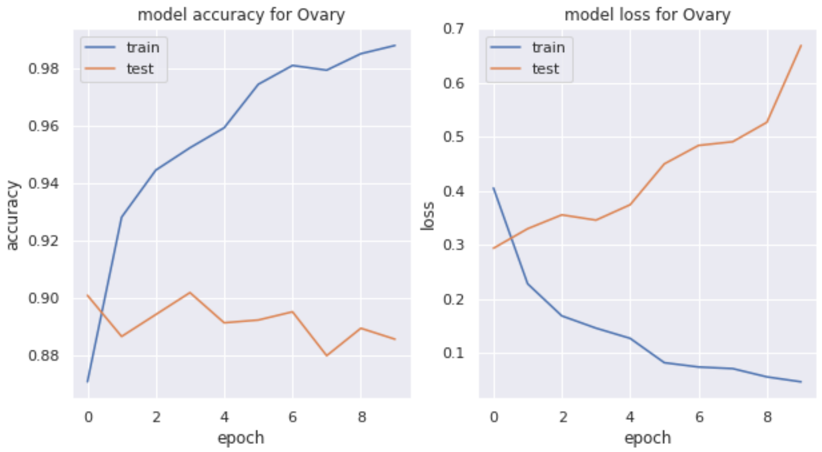

Figure 6: The accuracy and loss of the binary neural network for ovary.
