# Supplementary material for: Scalable analysis of multi-modal biomedical data
Source: Gigascience. 2021 Sep 11;10(9):giab058. doi: 10.1093/gigascience/giab058 (PMC8434767; doi:10.1093/gigascience/giab058)
Supplement: giab058_Response_to_Reviewer_Comments_Original_Submission [file giab058_response_to_reviewer_comments_original_submission.pdf]

Author's Response To Reviewer Comments

Close

** Note this is also provided in response.pdf **

Dear Editor and Reviewers,

Thank you all for taking the time to review our manuscript and provide the invaluable feedback. The goal of this manuscript was to describe our nested query compilation framework, how we have applied it to biomedical analysis, and the remaining architectural pieces that have enabled this application. We hope that our revised manuscript has provided a view of the architecture that is more accessible to users of varying backgrounds.
The next two sections will address each reviewer comment. We agree with all suggested revisions and have addressed each in the below sections, as well as in the main body of the paper. All revisions are highlighted blue in the main body of the paper. Revisions specific to each response are pointed out under the relevant comment.

Detailed response to comments of Reviewer 1

• While the topic and tool are interesting, the writing can be improved. The current manuscript reads more like a technical manual than a scientific paper. For example, in the background, the discussion on data modeling in the contexts of multi-omics analysis and distributed systems is extensive, but the writing can be better organized. The examples are helpful, but they are very technical and can be hard to follow. It would be good if the main challenges can be summarized on a high level. It might also be useful to have an example analysis use case to lead the technical discussion on data modeling.

Response:

We have summarized the main challenges on a high level as suggested, categorizing challenges as either related to performance or programming issues. We also center the discussion of each challenge around a running example. The goal was to defer much of the technical discussion until after the running example is overviewed informally, eventually reaching the query language and shredded representation where the application of the running example is presented.

• It is also unclear how are the targeted users of the tool and why distributed computing is needed. For example, in application 1 & 2, it is unclear why distributed computing is necessary.

Response:

We now discuss the targeted users in the introduction. We also clarify where issues of scale emerge in the introduction, specifically referring to the ability of
datasets collected overtime to increase to a scale that would not be feasible to compute on without distributed computing.

For application 1, we clarify that the framework and distributed processing is used for the heavy-lifting portion of integrating datasets to build feature matrices. The related performance metric for this application is now moved to the Scalability experiments section. Here, we clarify that we are using a single chromosome worth of data for only 2500 samples to measure performance on a smaller scale machine. Though we are already seeing performance advantages of the framework for this smaller-scale data, distributed computing will become even more necessary as we consider processing whole genomes and more samples. We have clarified this in the text. In summary, the goal of application 1 was to highlight performance benefits that would be relevant for building genomics-based feature sets; based on current learning pipelines, aggregating large-scale datasets can produce a more light-weight set of features that can then be operated on with in-memory statistical packages, such as those that python provides.

For Application 2, we now provide a scalability experiment that specifically highlights how distributed computing can speed up the runtime for the most expensive steps in the driver gene analysis pipeline. These results show that for a variety of cluster configurations, the performance of this analysis improves as distribution increases.

Detailed response to comments of Reviewer 2

• The high-level querying language supported by TraNCE is not original because, as far as I understand, it has been presented in a previous paper [1] (which has been written by almost the same authors and that has been correctly referenced to in this submission). Even the TraNCE framework is not completely original because its name appears as the name of the project containing the code presented in [1]. Finally, at least one of the experiments presented in [1] seems to have been run on the same Hadoop installation used for the experiments presented in the current submission, and has involved the same datasets from the International Cancer Genome Consortium. So, I am a bit confused about what it is original in this new submission and what has been borrowed from [1]. My advice is to definitely clarify this point.

Response:

We agree this was previously overlooked. The goal of this paper is to highlight the usage and extensions of the framework that are specific to large-scale biomedical data analyses. The initial work was presented to a highly technical audience where none of the interesting aspects of the biological datasets / analyses were discussed. We believe it is important to present the technical details and extensions to the biological community at a level that is accessible to a wider audience. In the revised version we have added a note in the Related Work section to clarify this. Additionally, in the resubmitted version there is no overlap in the experiment presented. We include a new experiment using the same datasets that highlight both scalability and skew.

• Another issue that I think should be addressed is about the proposed framework being scalable. The authors state that the framework supports scalable processing of complex datatypes, however, no evidence is brought about this claim. The several different experiments that are reported seem to focus more on the expressiveness of the proposed language while no experiment about the scalability of the generated code is provided when run on a computing architecture of increasing size. I think we may agree on the fact that using Spark does not means that your code is scalable, neither I think it is enough to say that the scalability of TraNCE has been proved in [1]. So, I would suggest to elaborate also on this.

Response:

We have added a separate set of experiments concerning scalability. The scalability experiment shows that performance improves with an increasing amount of resources; we present this for a variety of cluster configurations.

• To be honest, I am a bit skeptical about the practical performance of the standard compilation route. I think that when applied to very large datasets it is likely to return huge RDDs that could require very long processing times. Instead, the shredded compilation route looks much clever to me. Could you elaborate further on this difference, especially according to the results of your experimentations?

Response:

Flattening methods are the current state-of-the-art for the handling nested datasets. As such, we include the standard pipeline as a baseline metric to measure performance of flattening methods in order to compare to shredding within the same platform. In the revised manuscript, we have now clarified that the standard pipeline is relevant only as a baseline in both the description of the standard compilation route, as well as relevant portions of the experiments. In addition, the new experiments that we add solely focus on the shredded compilation route such that more importance and focus is placed on this optimization.

• I also disagree with your idea of not describing how data skewness is dealt with in your framework. It is indeed one of the main cause for bad performance of many distributed applications so it would be interesting to know how did you manage this problem in your particular case.

Response:

We have added more detail on skew-handling as well as a separate set of experiments. Our previous paper only discussed a single sampling strategy only in the context of simulated data. When we applied the skew-handling procedure to the biological datasets, we revealed some interesting aspects of a heuristic procedure that was not obvious before.

Overall, all of your comments have been very helpful in shaping this paper and we hope that we have appropriately addressed your suggested improvements. Thank you again for taking the time to provide feedback.

Sincerely,

Jaclyn Smith, Michael Benedikt, Milos Nikolic, and Yao Shi

Close
