# Supplementary material for: Scalable analysis of multi-modal biomedical data
Source: Gigascience. 2021 Sep 11;10(9):giab058. doi: 10.1093/gigascience/giab058 (PMC8434767; doi:10.1093/gigascience/giab058)
Supplement: giab058_Response_to_Reviewer_Comments_Revision_2 [file giab058_response_to_reviewer_comments_revision_2.pdf]

Author's Response To Reviewer Comments

Close

Reference edits have been made.

Close
