# Supplementary material for: Scalable analysis of multi-modal biomedical data
Source: Gigascience. 2021 Sep 11;10(9):giab058. doi: 10.1093/gigascience/giab058 (PMC8434767; doi:10.1093/gigascience/giab058)
Supplement: giab058_Reviewer_2_Report_Original_Submission — Umberto Ferraro Petrillo -- 3/3/2021 Reviewed [file giab058_reviewer_2_report_original_submission.pdf]

Reviewer Report

Title: Scalable Analysis of Multi-Modal Biomedical Data

Version: Original Submission **Date:** 3/3/2021

Reviewer name: Umberto Ferraro Petrillo

Reviewer Comments to Author:

The authors propose a new framework, called TraNCE, for automating the design of distributed analysis pipelines over complex biomedical data types. They focus on the problem of unrolling references between different datasets (which can be very large), assuming that these datasets contain complex data types consisting of structured objects containing collections of other objects. By using TraNCE, it is possible to formulate queries over collections of nested data using a very high-level declarative language. Then, these queries are translated by TraNCE in Apache Spark applications able to implement those queries in an efficient and scalable way. Apart from a quick description of the TraNCE framework and of the declarative language it supports, the paper also includes a vast collection of examples of multi-omics analyses conducted using TraNCE on real-world data.

I found the contribution proposed by this paper to be very actual. Indeed, there is a flourishing of public multi-omics databases. But, their huge volumes make their analysis difficult and very expensive, if not approached with the right methodologies. Distributed analysis frameworks like Spark can be of help, but they are often not easy to be mastered, especially for those not having deep distributed programming skills. So, TraNCE looks like a very much need contribution on this topic.

However, I have some remarks. The high-level querying language supported by TraNCE is not original because, as far as I understand, it has been presented in a previous paper [1] (which has been written by almost the same authors and that has been correctly referenced to in this submission). Even the TraNCE framework is not completely original because its name appears as the name of the project containing the code presented in [1]. Finally, at least one of the experiments presented in [1] seems to have been run on the same Hadoop installation used for the experiments presented in the current submission, and has involved the same datasets from the International Cancer Genome Consortium. So, I am a bit confused about what it is original in this new submission and what has been borrowed from [1]. My advice is to definitely clarify this point.

Another issue that I think should be addressed is about the proposed framework being scalable. The authors state that the framework supports scalable processing of complex datatypes, however, no evidence is brought about this claim. The several different experiments that are reported seem to focus more on the expressiveness of the proposed language while no experiment about the scalability of the generated code is provided when run on a computing architecture of increasing size. I think we may agree on the fact that using Spark does not means that your code is scalable, neither I think it is enough to say that the scalability of TraNCE has been proved in [1]. So, I would suggest to elaborate also on this.

To be honest, I am a bit skeptical about the practical performance of the standard compilation route. I think that when applied to very large datasets it is likely to return huge RDDs that could require very long processing times. Instead, the shredded compilation route looks much clever to me. Could you

elaborate further on this difference, especially according to the results of your experimentations? I also disagree with your idea of not describing how data skewness is dealt with in your framework. It is indeed one of the main cause for bad performance of many distributed applications so it would be interesting to know how did you manage this problem in your particular case.
On the bright side, I really appreciated the flexibility of the proposed framework, as witnessed by the vast amount of examples provided, as well as its positive implications on the analysis of multi-omics databases.
Finally, the English of the manuscript is very good and I have not been able to find any typos so far.
[1] Jaclyn Smith, Michael Benedikt, Milos Nikolic, and Amir Shaikhha. 2020. Scalable querying of nested data. Proc. VLDB Endow. 14, 3 (November 2020), 445-457.

Level of Interest

Please indicate how interesting you found the manuscript: Choose an item.

Quality of Written English

Please indicate the quality of language in the manuscript: Choose an item.

Declaration of Competing Interests

Please complete a declaration of competing interests, considering the following questions:

- Have you in the past five years received reimbursements, fees, funding, or salary from an organisation that may in any way gain or lose financially from the publication of this manuscript, either now or in the future?
- Do you hold any stocks or shares in an organisation that may in any way gain or lose financially from the publication of this manuscript, either now or in the future?
- Do you hold or are you currently applying for any patents relating to the content of the manuscript?
- Have you received reimbursements, fees, funding, or salary from an organization that holds or has applied for patents relating to the content of the manuscript?
- Do you have any other financial competing interests?
- Do you have any non-financial competing interests in relation to this paper?

If you can answer no to all of the above, write 'I declare that I have no competing interests' below. If your reply is yes to any, please give details below.

I declare that I have no competing interests

I agree to the open peer review policy of the journal. I understand that my name will be included on my report to the authors and, if the manuscript is accepted for publication, my named report including any attachments I upload will be posted on the website along with the authors' responses. I agree for my report to be made available under an Open Access Creative Commons CC-BY license (http://creativecommons.org/licenses/by/4.0/). I understand that any comments which I do not wish to be included in my named report can be included as confidential comments to the editors, which will not be published.

Choose an item.

To further support our reviewers, we have joined with Publons, where you can gain additional credit to further highlight your hard work (see: https://publons.com/journal/530/gigascience). On publication of this paper, your review will be automatically added to Publons, you can then choose whether or not to claim your Publons credit. I understand this statement.

Yes Choose an item.
